# Prevalence of Select New Symptoms and Conditions Among Persons Aged Younger Than 20 Years and 20 Years or Older at 31 to 150 Days After Testing Positive or Negative for SARS-CoV-2

**DOI:** 10.1001/jamanetworkopen.2021.47053

**Published:** 2022-02-04

**Authors:** Alfonso C. Hernandez-Romieu, Thomas W. Carton, Sharon Saydah, Eduardo Azziz-Baumgartner, Tegan K. Boehmer, Nedra Y. Garret, L. Charles Bailey, Lindsay G. Cowell, Christine Draper, Kenneth H. Mayer, Kshema Nagavedu, Jon E. Puro, Sonja A. Rasmussen, William E. Trick, Valentine Wanga, Jennifer R. Chevinsky, Brendan R. Jackson, Alyson B. Goodman, Jennifer R. Cope, Adi V. Gundlapalli, Jason P. Block

**Affiliations:** 1 Epidemic Intelligence Service, Centers for Disease Control and Prevention, Atlanta, Georgia; 2 COVID-19 Response, Centers for Disease Control and Prevention, Atlanta, Georgia; 3 Louisiana Public Health Institute, New Orleans, Louisiana; 4 Applied Clinical Research Center, Department of Pediatrics, Children’s Hospital of Philadelphia, Philadelphia, Pennsylvania; 5 Department of Population and Data Sciences, Department of Immunology, University of Texas Southwestern Medical Center, Dallas; 6 Department of Population Medicine, Harvard Pilgrim Health Care Institute, Harvard Medical School, Boston, Massachusetts; 7 Fenway Health, Boston, Massachusetts; 8 OCHIN Inc, Portland, Oregon; 9 Department of Pediatrics, University of Florida College of Medicine, Gainesville; 10 Health Research & Solutions, Cook County Health, Chicago, Illinois

## Abstract

**Importance:**

New symptoms and conditions can develop following SARS-CoV-2 infection. Whether they occur more frequently among persons with SARS-CoV-2 infection compared with those without is unclear.

**Objective:**

To compare the prevalence of new diagnoses of select symptoms and conditions between 31 and 150 days after testing among persons who tested positive vs negative for SARS-CoV-2.

**Design, Setting, and Participants:**

This cohort study analyzed aggregated electronic health record data from 40 health care systems, including 338 024 persons younger than 20 years and 1 790 886 persons aged 20 years or older who were tested for SARS-CoV-2 during March to December 2020 and who had medical encounters between 31 and 150 days after testing.

**Main Outcomes and Measures:**

*International Statistical Classification of Diseases, Tenth Revision, Clinical Modification *codes were used to capture new symptoms and conditions that were recorded 31 to 150 days after a SARS-CoV-2 test but absent in the 18 months to 7 days prior to testing. The prevalence of new symptoms and conditions was compared between persons with positive and negative SARS-CoV-2 tests stratified by age (20 years or older and young than 20 years) and care setting (nonhospitalized, hospitalized, or hospitalized and ventilated).

**Results:**

A total of 168 701 persons aged 20 years or older and 26 665 younger than 20 years tested positive for SARS-CoV-2, and 1 622 185 persons aged 20 years or older and 311 359 younger than 20 years tested negative. Shortness of breath was more common among persons with a positive vs negative test result among hospitalized patients (≥20 years: prevalence ratio [PR], 1.89 [99% CI, 1.79-2.01]; <20 years: PR, 1.72 [99% CI, 1.17-2.51]). Shortness of breath was also more common among nonhospitalized patients aged 20 years or older with a positive vs negative test result (PR, 1.09 [99% CI, 1.05-1.13]). Among hospitalized persons aged 20 years or older, the prevalence of new fatigue (PR, 1.35 [99% CI, 1.27-1.44]) and type 2 diabetes (PR, 2.03 [99% CI, 1.87-2.19]) was higher among those with a positive vs a negative test result. Among hospitalized persons younger than 20 years, the prevalence of type 2 diabetes (PR, 2.14 [99% CI, 1.13-4.06]) was higher among those with a positive vs a negative test result; however, the prevalence difference was less than 1%.

**Conclusions and Relevance:**

In this cohort study, among persons hospitalized after a positive SARS-CoV-2 test result, diagnoses of certain symptoms and conditions were higher than among those with a negative test result. Health care professionals should be aware of symptoms and conditions that may develop after SARS-CoV-2 infection, particularly among those hospitalized after diagnosis.

## Key Points

**Question:**

Are select new symptoms and conditions more common among persons aged younger than 20 years and 20 years or older who tested positive for SARS-CoV-2 compared with those who tested negative?

**Findings:**

In this cohort study of 338 024 persons younger than 20 years and 1 790 886 persons 20 years or older who were tested for SARS-CoV-2, new diagnoses of shortness of breath, nonspecific heart rate abnormalities, and type 2 diabetes were more common among those hospitalized after positive compared with negative test results; fatigue was more common among those aged 20 years or older.

**Meaning:**

Given these findings, health care professionals should be aware of new symptoms and conditions that may develop after SARS-CoV-2 infection, particularly among those hospitalized.

## Teaser

This cohort study examines the prevalence of select new symptoms and conditions diagnosed within 31 and 150 days after SARS-CoV-2 testing among persons who tested positive vs negative for the virus.

## Introduction

Understanding the frequency with which new symptoms and conditions emerge in the months following SARS-CoV-2 infection is important to inform patients’ expectations for recovery and allow health care professionals and health systems to address patients’ needs. Shortness of breath, fatigue or muscle weakness, and mild subjective cognitive dysfunction (ie, “brain fog”) are among the most commonly reported persistent symptoms in the months following SARS-CoV-2 infection.^1,2,3^ Studies have also documented an increase in diagnoses of new-onset neuropsychiatric conditions and type 2 diabetes among persons after SARS-CoV-2 infection.^4,5^ New symptoms and conditions months after a positive SARS-CoV-2 test have been described among small cohorts of nonhospitalized and hospitalized persons.^2,6,7^ However, population-based estimates of the occurrence of new symptoms and conditions following a diagnosis of SARS-CoV-2 infection remain undercharacterized in the US. In addition, whether certain new symptoms and conditions occur more frequently among persons with SARS-CoV-2 infection compared with those without has not been well established.

Use of electronic health records (EHRs) provides an opportunity to characterize the occurrence of symptoms and conditions among large populations. The National Patient-Centered Clinical Research Network, PCORnet, is a national-scale research network of analysis-ready longitudinal EHR data composed of more than 60 health care systems from all census regions of the US.^8^ Starting in April 2020, PCORnet began COVID-19 surveillance among 42 health care systems and 1 health plan with data available for approximately 9 million persons aged 20 years or older and nearly 3 million older than 20 years.

We used PCORnet data to examine the prevalence of select new symptoms and conditions in the 31 to 150 days after SARS-CoV-2 testing among persons aged younger than 20 and 20 years or older with positive and negative SARS-CoV-2 test results. Symptoms (eg, shortness of breath, fatigue, and cognitive dysfunction) and conditions (eg, type 2 diabetes and neurological disorders) were selected based on previous reports of SARS-CoV-2 infection sequelae.^1,2,3,4,5,9,10^ We aimed to (1) describe the prevalence of new symptoms and conditions diagnosed at medical encounters between 31 and 150 days after SARS-CoV-2 infection, stratified by age group and care setting, and (2) determine whether new symptoms and conditions were more common among persons with a positive test result for SARS-CoV-2 (hereafter referred to as “positive test”) compared with those with a negative test for SARS-CoV-2 (hereafter referred to as “negative test”). We hypothesized that (1) among persons with a positive test, symptoms and conditions would occur more frequently among those who received a higher level of care after diagnosis, and (2) if new symptoms and conditions were sequelae of SARS-CoV-2 infection, their prevalence would be higher among persons with a positive test.

## Methods

### PCORnet Data Model and Query

Descriptions of PCORnet are available elsewhere.^8,11^ Briefly, PCORnet uses a Common Data Model to facilitate data interoperability and centralized querying of longitudinal EHR data using modular statistical programs. Starting in April 2020, the network began supporting rapid querying (up to twice monthly), allowing for capture of data on patients tested for SARS-CoV-2 (identified using *Logical Observation Identifiers Names and Codes* [LOINC]). Queries were performed at each participating health care system using patient-level EHR data; results were transmitted to investigators in aggregated tabular format. This cohort study, included within a general SARS-CoV-2 surveillance project across PCORnet institutions, was deemed exempt from review under the public health surveillance provision of the Common Rule by the Harvard Pilgrim Health Care institutional review board. The design and analysis meets the Strengthening the Reporting of Observational Studies in Epidemiology (STROBE) reporting guideline.

**Figure 1.**
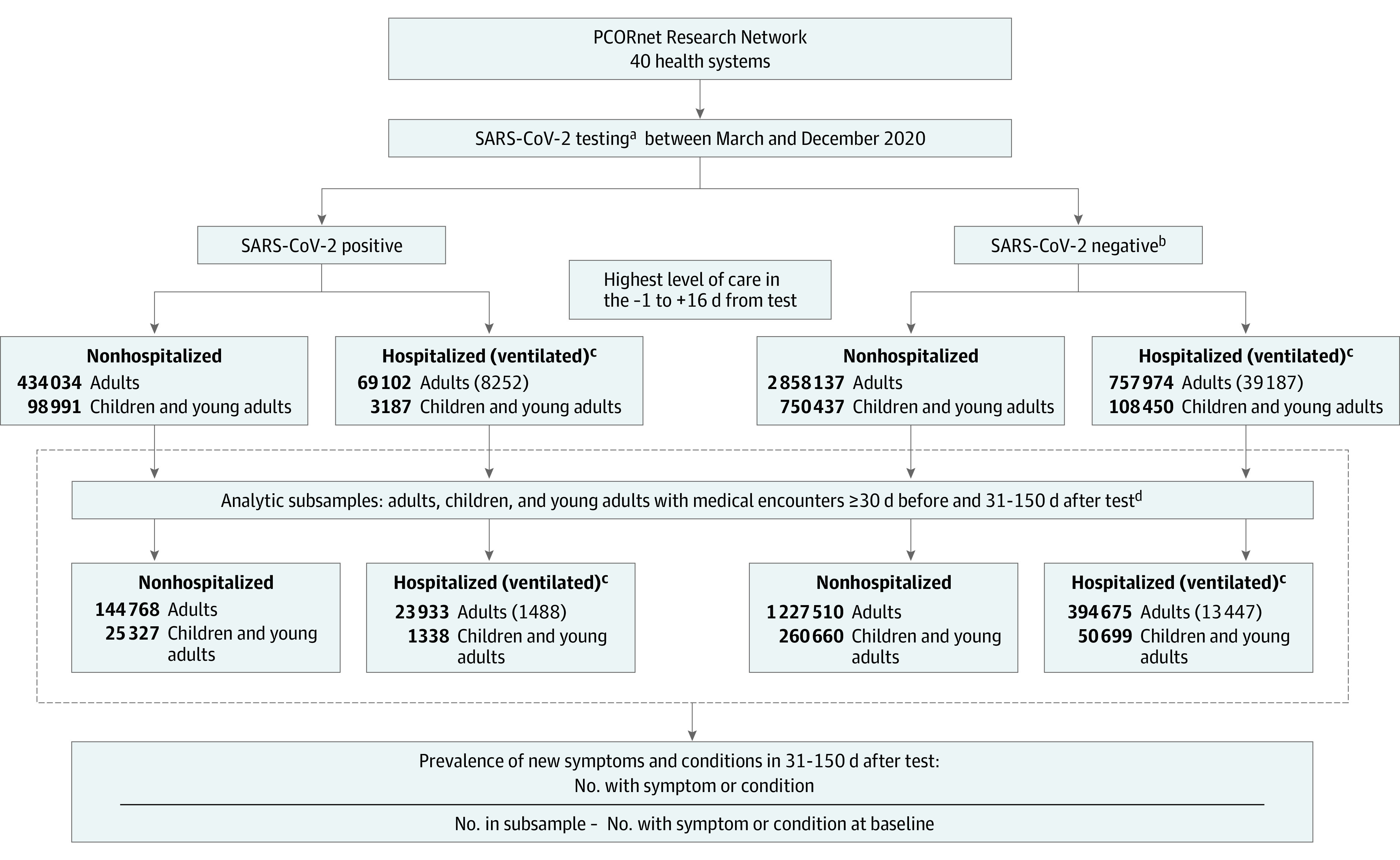
Flow Diagram Flow diagram of prevalence of new symptoms and conditions 31 to 150 days after SARS-CoV-2 infection between March and December 2020. Adults and children and young adults with positive and negative SARS-CoV-2 test results were stratified by care setting, and those with medical encounters 31 to 150 days after SARS-CoV-2 testing were analyzed. We compared prevalence of new symptoms and conditions between adults and children and young adults with positive and negative SARS-CoV-2 test results by care setting. ^a^Included all individuals with polymerase chain reaction (99%) or antigen (1%) testing. ^b^Patients never had a positive test result during the entire study period (ie, up to 150 days after SARS-CoV-2 test). ^c^Owing to the small sample of mechanically ventilated children with a positive SARS-CoV-2 test result, we did not assess new symptoms and conditions for this group. ^d^To ensure data completeness for symptoms and conditions, we restricted the analysis to patients who had at least 1 medical encounter in any care setting recorded 3 years to 1 month before and 31 to 150 days after SARS-CoV-2 testing. PCORnet indicates the National Patient-Centered Clinical Research Network.

In March 2021, 40 sites responded to a query assessing the occurrence of select symptoms and conditions diagnosed in the 31 to 150 days after SARS-CoV-2 testing; 2 sites were excluded because of data-quality issues and response delays (Figure 1; eTable 2 in the Supplement). The query included persons aged 20 years or older and younger than 20 years with SARS-CoV-2 polymerase chain reaction (99%) or antigen (1%) testing between March and December 2020 with follow-up until mid-March 2021 (Figure 1). We stratified age groups by care setting according to the highest level of care (ie, nonhospitalized, hospitalized, and hospitalized and ventilated) recorded between the day prior and 16 days after the index date of their first positive or negative test. Because of small sample sizes, we were unable to characterize a subgroup of ventilated persons younger than 20 years (n = 172). To maximize data completeness on new symptoms and conditions, we restricted the analysis to patients who had at least 1 medical encounter in any care setting recorded within 3 years to 30 days before and 31 to 150 days after SARS-CoV-2 testing (ie, connected to the health care system before and after SARS-CoV-2 testing). Patient-level data were stored behind institutional firewalls; the query returned only aggregate data for analysis. Information on race and ethnicity was abstracted directly from the EHR, which is typically self-reported by patients and captured when patients register for care at an institution.

### Patient Characteristics, Symptoms, and Conditions

We captured aggregate information on patient age, sex, race, ethnicity, and underlying symptoms and conditions up to 18 months prior to SARS-CoV-2 testing. Race categories included African American or Black, Asian, White, other (ie, American Indian or Alaska Native, Native Hawaiian or other Pacific Islander, or multiple race), and missing. Ethnicity categories included Hispanic, non-Hispanic, other, and missing. Symptoms and conditions among persons aged 20 years or older (n = 16) and younger than 20 years (n = 12) were defined using grouped *International Classification of Diseases, Tenth Revision, Clinical Modification* (*ICD-10-CM*) codes (eTable 1 in the Supplement). We included all *ICD-10-CM* codes for these symptoms and conditions recorded in encounters 31 to 150 days after SARS-CoV-2 testing. Persons with a single *ICD-10-CM* code (eg, shortness of breath) recorded in the 31 to 150 days were only counted once; however, patients with multiple symptoms or conditions of interest were counted separately for each symptom or condition of interest.

### Group-Level Prevalence of New Symptoms and Conditions

To assess prevalence of new symptoms and conditions, we specified 3 time periods relative to the index date of SARS-CoV-2 test: 18 months to 7 days before testing (baseline), 6 days before to 30 days after testing (short-term), and 31 to 150 days after testing (long-term). We set the index date as the date of the first positive or negative test; the cohort of patients with negative tests never had a positive test during the entire query period (ie, up to 150 days after first SARS-CoV-2 test). New symptoms and conditions were defined as those present in the long-term period and absent in the baseline period. Prevalence was calculated by dividing the number of persons having a given symptom or condition in the long-term phase but not at baseline by the total persons in the cohort minus the number of persons with a given symptom or condition in the baseline period (Figure 1).

### Statistical Analysis

Persons with a positive and negative test were divided into cohorts by age (20 years or older and younger than 20 years) and care setting. Within cohorts, we compared the distribution of age, sex, race, ethnicity, and underlying conditions using χ^2^ tests. We used a Bonferroni correction to adjust for multiple testing, and statistical significance was considered *P* < .0008. We were unable to perform multivariable adjustment for differences in demographic characteristics, underlying conditions, and follow-up because of the aggregated format of the data. We compared the prevalence of new symptoms and conditions between persons with a positive and negative test within age and care setting cohorts using unadjusted prevalence ratios (PRs) with 99% CIs. Exclusion of the null (PR of 1.0), rather than *P *values, was used to determine whether PRs were significantly different between persons with a positive and a negative test.

## Results

### Population Characteristics

During March to December 2020, among 1 790 886 persons aged 20 years or older (694 751 men, 1 096 039 women, and 96 other or missing sex) who had medical encounters in the 31- to 150-day period after index (henceforth referred to as medical encounters), we identified 144 768 nonhospitalized and 23 933 hospitalized (of whom 1488 were ventilated) persons with a positive test, and 1 227 510 nonhospitalized and 394 675 hospitalized (13 447 ventilated) persons with a negative test. Among 338 024 persons younger than 20 years (170 206 male, 167 805 female, and 13 other or missing sex) with medical encounters, we identified 25 327 nonhospitalized and 1338 hospitalized persons with a positive test and 260 660 nonhospitalized and 50 699 hospitalized persons with a negative test (Table 1). Among both age cohorts, the proportion of persons with medical encounters was higher among those with a negative test compared with those with a positive test. Persons younger than 20 years with positive tests were older, with more who were aged 13 to younger than 20 years, than those with negative tests (49% vs 35%). More persons aged 20 years or older (18%) and younger than 20 years (26%) with positive tests were Hispanic across all care settings compared with those with negative tests (8% and 14%, respectively) (Table 1).

**Table 1.** Demographic and Clinical Characteristics of Adults and Children and Young Adults With a Positive or Negative SARS-CoV-2 Test Result With Medical Encounters 31 to 150 Days After First Test

Characteristic	No. (%)^a^									
	Adults						Children and young adults			
	Non-hospitalized		Hospitalized		Mechanically ventilated		Non-hospitalized		Hospitalized	
	Positive	Negative	Positive	Negative	Positive	Negative	Positive	Negative	Positive	Negative
No. with a SARS-CoV-2 test during March-December 2020	434 034	2 858 137	69 102	757 974	8252	39 187	98 991	750 437	3187	108 450
Had a medical encounter 31-150 d after test	144 768 (33)	1 227 510 (43)	23 933 (35)	394 675 (52)	1488 (18)	13 447 (34)	25 327 (26)	260 660 (35)	1338 (42)	50 699 (47)
Age group, y										
0-<1	NA	NA	NA	NA	NA	NA	2196 (9)	24 222 (9)	157 (12)	5943 (12)
1-<6	NA	NA	NA	NA	NA	NA	4811 (19)	79 300 (30)	262 (20)	13 051 (26)
6-<13	NA	NA	NA	NA	NA	NA	5904 (23)	68 726 (26)	251 (19)	12 306 (24)
13-<20	NA	NA	NA	NA	NA	NA	12 405 (49)	88 412 (34)	668 (50)	19 382 (38)
20-39	48 535 (34)	340 387 (28)	3945 (16)	92 942 (24)	160 (11)^b^	1819 (14)^b^	NA	NA	NA	NA
40-54	39 892 (28)	289 010 (24)	4562 (19)	70 721 (18)	339 (23)	2581 (19)	NA	NA	NA	NA
45-64	27 321 (19)	244 673 (20)	5153 (22)	78 988 (20)	416 (28)	3611 (27)	NA	NA	NA	NA
≥65	29 020 (20)	353 440 (29)	10 263 (43)	152 024 (39)	565 (38)	5436 (40)	NA	NA	NA	NA
Sex										
Female	91 454 (63)	760 505 (62)	12 877 (54)	231 203 (59)	661 (44)^b^	5589 (42)^b^	13 257 (52)	128 492 (49)	686 (51)^b^	25 370 (50)^b^
Male	53 307 (37)	466 936 (38)	11 054 (46)	163 454 (41)	827 (56)	7857 (58)	12 070 (48)	132 157 (51)	652 (49)	25 327 (50)
Ethnicity										
Hispanic	25 144 (17)	105 333 (9)	4457 (19)	32 514 (8)	286 (19)	851 (6)	6643 (26)	37 169 (14)	408 (30)	7072 (14)
Non-Hispanic	113 975 (79)	1 063 976 (87)	18 837 (79)	350 387 (89)	1149 (77)	12 296 (91)	17 885 (71)	213 528 (82)	894 (67)	42 550 (84)
Other^c^	831 (1)	11 341 (1)	25 (0)	401 (0)	0	20 (0)	84 (0)	1106 (0)	0	67 (0)
Missing	4818 (3)	46 860 (4)	601 (3)	11 363 (3)	0	272 (2)	702 (3)	8853 (3)	0	994 (2)
Race										
African American or Black	22 982 (16)	185 734 (15)	6558 (27)	72 342 (18)	463 (31)	2699 (20)	4016 (16)	37 102 (14)	375 (28)	9470 (19)
Asian	4102 (3)	32 716 (3)	593 (2)	9772 (2)	46 (3)	264 (2)	721 (3)	6944 (3)	35 (3)	1440 (3)
White	98 429 (68)	890, 454 (73)	12 503 (52)	275 284 (70)	683 (46)	9342 (69)	15 488 (61)	175 365 (67)	541 (40)	31 288 (62)
Other^d^	12 252 (8)	75 108 (6)	3127 (13)	26 776 (7)	217 (15)	885 (7)	3159 (12)	25 527 (10)	262 (20)	5706 (11)
Missing	6979 (5)	43 498 (4)	1125 (5)	10 488 (3)	67 (5)	246 (2)	1898 (7)	15 704 (6)	125 (9)	2770 (5)
Underlying conditions^e^										
Hypertension	48 337 (33)	435 852 (36)	13 568 (57)	176 757 (45)	850 (57)^b^	7801 (58)^b^	NA	NA-	NA	NA
Type 2 diabetes	24 062 (17)	189 378 (15)	8193 (34)	82 052 (21)	567 (38)	3938 (29)	140 (1)	948 (0)	45 (3)	492 (1)
Chronic pulmonary disorders	20 083 (14)	207 514 (17)	5479 (23)	76 089 (19)	332 (22)	3773 (28)	2852 (11)^b^	30 211 (12)^b^	218 (16)^b^	6677 (13)^b^
Severe obesity (BMI ≥ 40)	12 951 (9)	98 309 (8)	3182 (13)	38 241 (10)	243 (16)	1678 (12)	NA	NA	NA	NA
Cancer	10 510 (7)	139 022 (11)	3046 (13)	68 429 (17)	149 (10)	2490 (19)	311 (1)	5415 (2)	135 (10)	2802 (6)
Coronary artery disease	9958 (7)	118 745 (10)	4610 (19)	64 788 (16)	284 (19)	3913 (29)	NA	NA	NA	NA
Chronic kidney disease	9033 (6)	95 183 (8)	5087 (21)	54 448 (14)	342 (23)^b^	2857 (21)^b^	NA	NA	NA	NA
Congestive heart failure	6676 (5)	81 657 (7)	4025 (17)	51 953 (13)	242 (16)	3386 (25)	NA	NA	NA	NA

Abbreviations: BMI, body mass index (calculated as weight in kilograms divided by height in meters squared); NA, not applicable.

^a^ Percentages may add to more than 100% because of rounding. Some percentages may add up to less than 100% because small cell sizes (n < 11) are suppressed at the time of reporting from sites or during data aggregation. Suppressed small cell sizes may result in differences between the overall cohort counts and the sum of stratified counts. The small cell sizes are presented as 0 values.

^b^ *P* > .0008, denoting nonsignificance. All comparisons not denoted by this footnote had a *P* < .0008. A *P* value for significance of .0008 was selected after adjusting for multiple comparisons using the Bonferroni method (α .05/60 hypothesis tests). Categories were compared using χ^2^ tests.

^c^ As specified by the PCORnet common Data Model specifications, the “other” category for ethnicity is used by institutions if race and ethnicity are listed together rather than separately.

^d^ Includes American Indian or Alaska Native, Native Hawaiian or other Pacific Islander, or multiple races.

^e^ Conditions diagnosed in the 18 months to 7 days prior to SARS-CoV-2 testing.

Type 2 diabetes and severe obesity were more common among persons aged 20 years or older with a positive test relative to those with a negative test across all care settings during baseline (18 months to 7 days prior to SARS-CoV-2 testing) (Table 1). Hospitalized persons younger than 20 years who tested positive had a higher proportion of cancer compared with those who tested negative (10% vs 6%) during baseline.

### Prevalence of New Symptoms Among Persons Younger Than 20 Years And 20 Years or Older With Positive Tests

Among persons aged 20 years or older with a positive test, shortness of breath (4.5% nonhospitalized, 10.5% hospitalized, and 16.6% ventilated), fatigue (4.2% non-hospitalized, 8.0% hospitalized, and 19.8% ventilated), and sleep disorders (3.2% nonhospitalized, 5.6% hospitalized, and 10.4% ventilated) were the most prevalent diagnoses (Table 2). Among persons aged younger than 20 years with a positive test, change in bowel habits (2.3% nonhospitalized and 6.0% hospitalized), fatigue (1.7% nonhospitalized and 3.5% hospitalized), and shortness of breath (1.8% nonhospitalized and 3.9% hospitalized) were the most prevalent diagnoses.

**Table 2.** Prevalence of New Diagnoses of Symptoms and Conditions Among Adults and Children and Young Adults With Positive and Negative SARS-CoV-2 Test Results With Medical Encounters 31 to 150 Days After First Test

Incident symptoms and conditions	No./total No. (%)^a^					
	Nonhospitalized		Hospitalized		Mechanically ventilated	
	Positive	Negative	Positive	Negative	Positive	Negative
**Adults**						
Symptoms						
Shortness of breath	5703/12 8251 (4.5)	43 208/1 061 692 (4.1)	1932/18 419 (10.5)	17 782/320 902 (5.5)	186/1123 (16.6)	896/9166 (9.8)
Fatigue	5300/125 749 (4.2)	47 017/1 061 342 (4.4)	1570/19 677 (8.0)	19 790/334 893 (5.9)	245/1236 (19.8)	1083/10 825 (10.0)
Sleep disorders	3857/122 613 (3.2)	38 835/101 3807 (3.8)	1058/18 821 (5.6)	16 124/322 673 (5.0)	118/1136 (10.4)	937/10 072 (9.3)
Headache	3682/131 406 (2.8)	33 758/1 125 058 (3.0)	517/22 162 (2.3)	8841/366 957 (2.4)	28/1404 (2.0)	323/12 716 (2.5)
Heart rate abnormality^b^	3509/133 276 (2.6)	29 543/1 114 330 (2.7)	1102/20 594 (5.4)	14 815/347 379 (4.3)	127/1303 (9.8)	865/10 964 (7.9)
Cognitive dysfunction	3267/131 245 (2.5)	31 989/1 099 309 (2.9)	1102/19 930 (5.5)	16 247/343 265 (4.7)	124/1251 (9.9)	949/11 129 (8.5)
Change in bowel habits	2733/132 350 (2.1)	29 966/1 103 068 (2.7)	879/20 657 (4.3)	15 365/346 939 (4.4)	96/1297 (7.4)	741/11 453 (6.5)
Weight loss	1132/140 719 (0.8)	16 511/1 177 543 (1.4)	848/22 331 (3.8)	13 605/367 617 (3.7)	167/1371 (12.2)	1034/11 757 (8.8)
Sensation and perception	1191/139 605 (0.9)	13 089/1 175 316 (1.1)	329/22 566 (1.5)	5806/375 507 (1.6)	25/1420 (1.8)	253/12 662 (2.0)
Conditions						
Anxiety or depression	3934/119 625 (3.3)	40 397/982 068 (4.1)	930/19 218 (4.8)	17 428/318 711 (5.5)	114/1197 (9.5)	885/10 477 (8.5)
Type 2 diabetes	1999/120 706 (1.7)	19 012/103 8132 (1.8)	1140/15 740 (7.2)	11 179/312 623 (3.6)	154/921 (16.7)	708/9509 (7.5)
Peripheral nerve disorders	1645/136 554 (1.2)	18 258/1 143 447 (1.6)	461/21 948 (2.1)	8049/364 039 (2.2)	99/1381 (7.2)	432/12 345 (3.5)
Ataxia or trouble walking	1004/140 466 (0.7)	12 979/1 177 461 (1.1)	496/22 218 (2.2)	8131/370 308 (2.2)	102/1398 (7.3)	443/12 493 (3.6)
Autonomic dysfunction	491/142 854 (0.3)	5928/1 205 120 (0.5)	231/23 265 (1.0)	4133/383 802 (1.2)	24/1459 (1.6)	230/12 897 (1.8)
Seizures	251/142 809 (0.2)	3498/1 206 410 (0.3)	208/23 143 (1.0)	3091/383 098 (0.8)	21/1418 (1.5)	378/12 656 (3.0)
Myoneural disorders	151/144 129 (0.1)	1556/1 220 689 (0.1)	148/23 667 (0.6)	1157/391 284 (0.3)	83/1455 (5.7)	130/13 184 (1.0)
**Children and young adults**						
Symptoms						
Change in bowel habits	523/22 999 (2.3)	6010/231 668 (2.6)	62/1040 (6.0)	2253/41 498 (5.4)	NA	NA
Fatigue	405/24 238 (1.7)	4033/249 755 (1.6)	42/1217 (3.5)	1191/47 439 (2.5)	NA	NA
Shortness of breath	426/24 049 (1.8)	3433/244 651 (1.4)	46/1179 (3.9)	1048/46 092 (2.3)	NA	NA
Headache	436/24 146 (1.8)	4303/251 359 (1.7)	19/1267 (1.5)	801/48 392 (1.7)	NA	NA
Heart rate abnormality^b^	251/24 545 (1.0)	2248/250 999 (0.9)	49/1139 (4.3)	1353/46 288 (2.9)	NA	NA
Cognitive dysfunction	283/24 388 (1.2)	2638/251 165 (1.1)	27/1243 (2.1)	880/47 769 (1.8)	NA	NA
Weight loss	169/24 586 (0.7)	1959/250 073 (0.8)	34/1191 (2.9)	1330/46 225 (2.9)	NA	NA
Sensation and perception	194/24 603 (0.8)	2969/248 714 (1.2)	17/1230 (1.4)	1143/46 891 (2.4)	NA	NA
Conditions						
Anxiety or depression	584/23 016 (2.5)	5196/240 107 (2.2)	53/1152 (4.6)	1772/45 258 (3.9)	NA	NA
Seizures	36/24 874 (0.1)	944/252 135 (0.4)	17/1203 (1.4)	899/45 667 (2.0)	NA	NA
Autonomic dysfunction	46/25 195 (0.2)	574/258 318 (0.2)	10/1309 (0.8)	364/49 574 (0.7)	NA	NA
Type 2 diabetes	27/25 187 (0.1)	220/259 712 (0.1)	17/1293 (1.3)	308/50 207 (0.6)	NA	NA

^a^ No. = Number of persons with the given symptom or condition diagnosed 31 to 150 days after SARS-CoV-2 testing who did not have the condition recorded in the 18 months to 7 days prior to SARS-CoV-2 testing; total No. = number of persons with medical encounters 31 to 150 days after SARS-CoV-2 testing minus the number of persons with medical encounters in 31 to 150 days who had the symptom or condition recorded in the 18 months to 7 days prior to SARS-CoV-2 testing. Total denominators are different for each row owing to removal of persons with symptoms and conditions in the baseline period.

^b^ Nonspecific heart rate abnormalities include tachycardia, bradycardia, and palpitations.

### Differences in Prevalence of New Symptoms Between Persons Younger Than 20 Years And 20 Years or Older With Positive and Negative Tests

**Figure 2.**
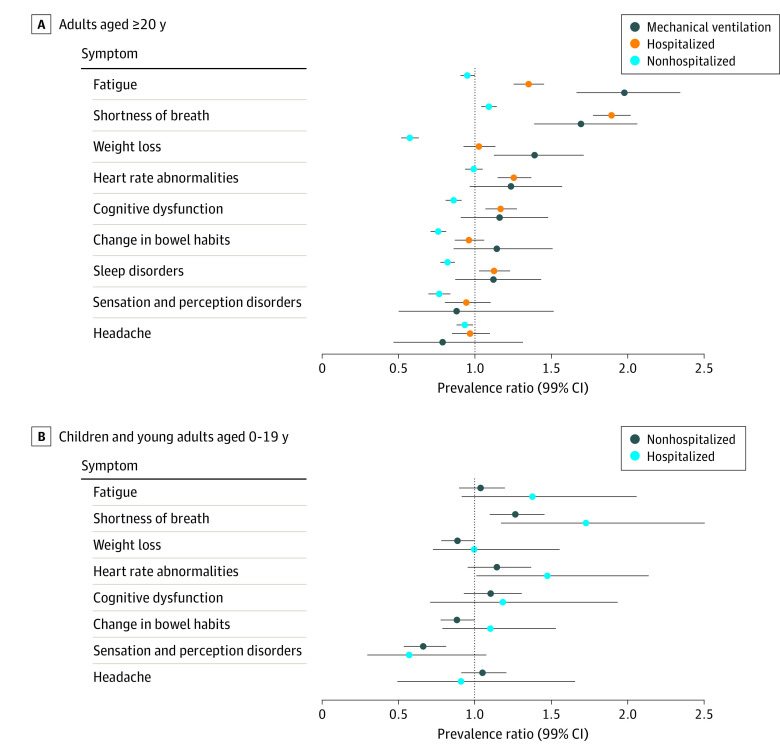
Prevalence Ratios of New Symptoms Among Adults and Children and Young Adults With Medical Encounters 31 to 150 Days After a First SARS-CoV-2 Test Between March and December 2020 Mechanically ventilated children were not included owing to the small sample size of children with a positive SARS-CoV-2 test result in this group (n = 172). Prevalence ratios are calculated as the proportion patients with a symptom who had a positive SARS-CoV-2 test result divided by the proportion of patients with a symptom who had a negative test result. Point estimates and 99% CIs (indicated by whiskers) are provided.

Among nonhospitalized persons aged 20 years or older, the prevalence of shortness of breath in the 31- to 150-day period was higher among those with a positive test compared with those with a negative test (PR, 1.09 [99% CI, 1.05-1.13]), whereas the prevalence of other symptoms was higher among persons with a negative test (Figure 2; eTable 3 in the Supplement). Among nonhospitalized persons younger than 20 years, new diagnoses of shortness of breath were more prevalent among those with positive tests compared with those with negative tests (PR, 1.26 [99% CI, 1.11-1.44]). (Figure 2; eTable 3 in the Supplement). However, for both age cohorts, prevalence differences were smaller than 1%.

In both age cohorts, among hospitalized persons with a positive test, the prevalence of shortness of breath (≥20 years: PR, 1.89 [99% CI, 1.79-2.01]; <20 years: PR, 1.72 [99% CI, 1.17-2.51]) and nonspecific heart rate abnormalities (ie, tachycardia, bradycardia, or palpitations) (≥20 years: PR 1.25 [1.16-1.36]; <20 years: PR 1.47 [1.02-2.12]) was significantly higher compared with those with a negative test (Figure 2; eTable 3 in the Supplement). Fatigue (PR, 1.35 [99% CI 1.27-1.44]), sleep disorders (PR, 1.12 [99% CI 1.04-1.22]), and cognitive dysfunction (PR, 1.17 [99% CI 1.08-1.26]) were higher among hospitalized persons aged 20 years or older with a positive compared with a negative test; however, absolute differences in prevalence were smaller than 1% for sleep disorders and cognitive dysfunction. Differences in symptom prevalence between persons aged 20 years or older with a positive vs negative test were greatest among those who were ventilated; prevalence of fatigue and shortness of breath were 10% and 7% higher among those with a positive test, respectively (Table 2).

### Prevalence of New Conditions Among Persons Younger Than 20 Years and 20 Years or Older With Positive Tests

The prevalence of new conditions among nonhospitalized persons aged 20 years or older with a positive test was less than 2% (Table 2). Among hospitalized persons aged 20 years or older with a positive test, type 2 diabetes (7.2%), anxiety and depression (4.9%), and ataxia or trouble walking (2.2%) were the most prevalent new diagnoses. Among ventilated persons aged 20 years or older with a positive test, prevalence was highest for type 2 diabetes (16.7%) and anxiety and depression (9.5%). New-onset peripheral nerve disorders (7.2%), ataxia and trouble walking (7.3%), and myoneural disorders (5.7%) were common among ventilated persons aged 20 years or older with a positive test, although these conditions were rare among their nonventilated hospitalized and nonhospitalized counterparts (≤2.5% prevalence). Among persons younger than 20 years with a positive test, anxiety and depression (2.6% nonhospitalized and 4.6% hospitalized) were the most prevalent new diagnoses.

### Differences in Prevalence of New Conditions Between Persons Younger Than 20 Years and 20 Years or Older With Positive and Negative Tests

**Figure 3.**
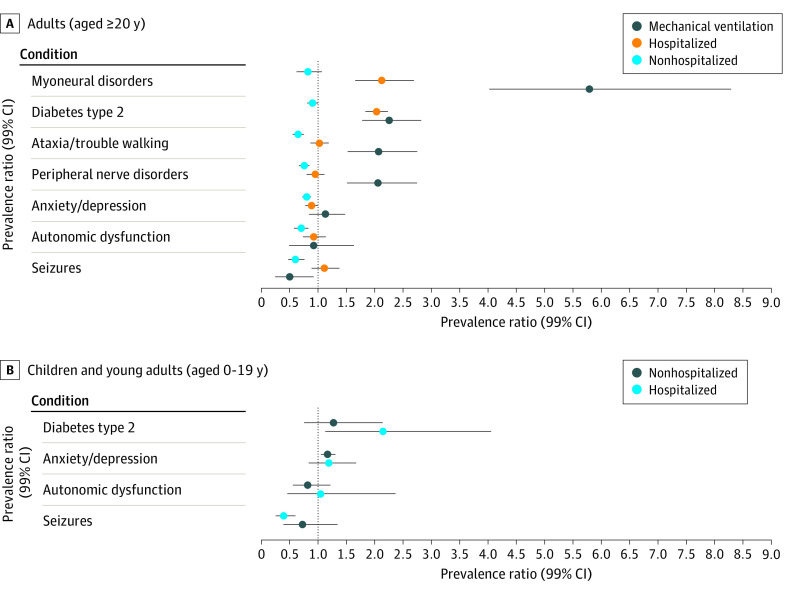
Prevalence Ratios of New Conditions Among Adults and Children and Young Adults With Medical Encounters 31 to 150 Days After a First SARS-CoV-2 Test Between March and December 2020 Mechanically ventilated children were not included owing to the small sample size of children with a positive SARS-CoV-2 test result in this group (n = 172). Prevalence ratios are calculated as the proportion patients with a condition who had a positive SARS-CoV-2 test result divided by the proportion of patients with a condition who had a negative test result. Point estimates and 99% CIs (indicated by whiskers) are provided.

Among nonhospitalized persons aged 20 years or older, several new conditions were more prevalent among those with a negative test compared with those with a positive test; however, prevalence differences were less than 1%. Among nonhospitalized persons younger than 20 years, the prevalence of anxiety and depression (PR, 1.17 [99% CI, 1.05-1.31]) was higher among those with positive vs a negative test (Figure 3; eTable 3 in the Supplement).

Among hospitalized persons aged 20 years or older, the prevalence of new type 2 diabetes (PR, 2.03 [99% CI, 1.87-2.19]) was significantly higher among those with a positive test compared with those with a negative test (Figure 3; eTable 3 in the Supplement). Among ventilated persons aged 20 years or older with a positive test, the prevalence of new type 2 diabetes (PR, 2.25 [99% CI, 1.82-2.77]), peripheral nerve disorders (PR, 2.05 [99% CI, 1.55-2.70]), ataxia and trouble walking (PR, 2.06 [99% CI, 1.57-2.70]), and myoneural disorders (PR, 5.79 [99% CI, 4.06-8.25]) were significantly higher compared with those with a negative test. Among hospitalized persons younger than 20 years, the prevalence of new type 2 diabetes (PR, 2.14 [99% CI, 1.13-4.06]) was higher among those with a positive test compared with those with a negative test; however, the prevalence difference was smaller than 1%.

## Discussion

In this cohort study, using EHR data from 168 701 persons aged 20 years or older and 26 665 aged younger than 20 years who tested positive for SARS-CoV-2, we observed that symptoms and conditions reported as possible sequelae of SARS-CoV-2 infection occurred infrequently (≤11% prevalence) in nonventilated persons with medical encounters at 31 to 150 days after SARS-CoV-2 testing. New diagnoses of shortness of breath were significantly higher among persons with a positive test compared with those with a negative test in both age cohorts. Diagnoses of fatigue, cognitive dysfunction, sleep disorders, heart rate abnormalities, myoneural disorders, and type 2 diabetes among hospitalized persons aged 20 years or older and heart rate abnormalities and type 2 diabetes among hospitalized persons younger than 20 years were more common for those with a positive vs negative test. Although new symptoms and conditions occurred infrequently, applying the proportions of these rare events to the millions of persons infected with SARS-CoV-2 means that a substantial number might experience new symptoms and conditions after their acute illness. In addition, new symptoms can be long-lasting^12^ and involve chronic conditions, such as type 2 diabetes. Increasing awareness of new symptoms and conditions among health care professionals and health systems is paramount to meet the needs of patients with prolonged or chronic sequelae of SARS-CoV-2 infection.

The prevalence of new symptoms and condition diagnoses following a positive test was higher among hospitalized persons compared with their nonhospitalized counterparts; new symptoms and conditions also were most prevalent among ventilated persons aged 20 years or older. Although the primary reason for hospitalization following SARS-CoV-2 testing was not available for this study, this study suggests that long-term symptoms and conditions may be more common with increased SARS-CoV-2 infection severity. Previous reports have highlighted a higher incidence of neuropsychiatric conditions and diabetes among hospitalized and ventilated adults with SARS-CoV-2 infection compared with those without.^4,5,13^ The higher prevalence of anxiety and depression, myopathies, peripheral nerve disorders, and weight loss among ventilated persons highlights the role of post–intensive care syndrome in compounding the effects of SARS-CoV-2 infection.^14^ Symptom prevalence among nonhospitalized persons aged 20 years or older in our study was similar to other studies that have found a prevalence of selected symptoms of less than 10% in the 6 months following SARS-CoV-2 infection.^6^ Similar to our findings, no increased risk for new symptoms and conditions in the postacute phase, other than shortness of breath and venous thromboembolism, was observed in a large population-based study of nonhospitalized adults with SARS-CoV-2 infection.^15^ Whether these differences were due to underlying demographic differences in patients infected with SARS-CoV-2 (compared with those who were not), differences in the types of care received (eg, aggressive medical treatment when hospitalized), or direct effects of the SARS-CoV-2 virus is unknown. Longitudinal studies extending beyond 6 months after acute infection will be important to quantify the true impact of COVID-19 sequelae.

The prevalence of new-onset diabetes in our population may be overestimated, as hyperglycemia or receipt of glucocorticoids during the acute event might have unmasked diabetes that was only coded during a follow-up visit when persistent hyperglycemia was recognized. Nevertheless, there is evidence of direct cytopathic effects of SARS-CoV-2 infection in pancreatic tissue and increases in insulin resistance owing to inflammation.^16,17^ Future studies should evaluate whether evidence for type 2 diabetes persists beyond the time period that could be affected by treatments.

Data regarding postacute symptoms and conditions in children are scarce. Studies have highlighted prolonged fatigue, headaches, sleep disturbances, cognitive dysfunction, and sensory disturbances in this population.^9,10,18^ In our study, shortness of breath and nonspecific heart rate abnormalities were significantly higher among hospitalized persons younger than 20 years who tested positive for SARS-CoV-2 compared with those who tested negative, whereas minimal differences were observed between these groups when not hospitalized. Our data suggest that, similar to adults, the risk of selected new symptoms and conditions among nonhospitalized persons younger than 20 years may be low.

### Limitations

There are several limitations to our study. First, use of diagnostic codes may have led to an underestimation of prevalent symptoms and conditions, as we only observed patients when they presented for care, and coding for illnesses and symptoms may not be comprehensive during visits. In addition, we defined *ICD-10-CM* code groups broadly based on reviews of the medical literature and clinical knowledge, which may have resulted in including symptoms and conditions unrelated to SARS-CoV-2 infection. Second, we defined new symptoms or conditions as the occurrence of 1 diagnostic code during the 31- to 150-day period to enhance sensitivity for detection of possible SARS-CoV-2 sequelae. Using 2 or more occurrences of diagnostic codes may have enhanced specificity; however, given that patients may have not had the opportunity for additional visits during the 120-day follow-up period, we selected enhanced sensitivity. Third, we were unable to determine whether new symptoms and conditions were due to effects of SARS-CoV-2 infection or the result of recognition of previously undiagnosed health problems. Fourth, we were unable to control for differences in demographic characteristics, underlying conditions, and follow-up between persons with positive and negative tests. Fifth, we used care setting following SARS-CoV-2 testing as a proxy for COVID-19 severity, which may have resulted in misclassification if persons with a positive test were hospitalized for reasons other than acute SARS-CoV-2 illness. Sixth, we were unable to ascertain whether SARS-CoV-2 testing was conducted because of symptoms, as a part of routine surveillance, or for travel purposes. Hospitalized persons who tested negative for SARS-CoV-2 included those hospitalized for nonviral illness (eg, pregnancy, trauma, chronic conditions) and may have biased our estimates, as there may be multiple etiologies for symptoms and conditions examined in this report. Finally, not all individuals in the study had 150 days of follow-up, which may have led to an underestimation of new symptoms and conditions among persons who tested positive in the months of November and December 2020.

## Conclusions

In this cohort study, we determined the prevalence of select new symptoms and conditions in a large health care–seeking population, highlighting symptoms and conditions that occurred more frequently among persons with a positive compared to those with a negative SARS-CoV-2 test, and found an increase in symptoms and conditions in those with higher severity of acute SARS-CoV-2 infection. These estimates highlight the need for health care professionals and patients to monitor for development of new symptoms and conditions beyond the first month after SARS-CoV-2 infection, particularly for individuals who required hospitalization for acute COVID-19.

## Notes

Supplement. **eTable 1.** New symptom and condition ICD-10-CM diagnostic codes **eTable 2.** Institutions contributing data **eTable 3.** Prevalence of new diagnoses of symptoms and conditions among adults and children and young adults with positive or negative SARS-CoV-2 test results with medical encounters 31 to 150 days after first test, PCORnet, March to December 2020
