# Supplementary material for: Prevalence of Select New Symptoms and Conditions Among Persons Aged Younger Than 20 Years and 20 Years or Older at 31 to 150 Days After Testing Positive or Negative for SARS-CoV-2
Source: JAMA Netw Open. 2022 Feb 4;5(2):e2147053. doi: 10.1001/jamanetworkopen.2021.47053 (PMC8817203; doi:10.1001/jamanetworkopen.2021.47053)
Supplement: Supplement. — eTable 1. New symptom and condition ICD-10-CM diagnostic codes eTable 2. Institutions contributing data eTable 3. Prevalence of new diagnoses of symptoms and conditions among adults and children and young adults with positive or negative SARS-CoV-2 test results with medical encounters 31 to 150 days after first test, PCORnet, March to December 2020 [file jamanetwopen-e2147053-s001.pdf]

Supplemental Online Content

Hernandez-Romieu AC, Carton TW, Saydah S, et al. Prevalence of select new symptoms and conditions among persons aged younger than 20 years and 20 years or older at 31 to 150 days after testing positive or negative for SARS-CoV-2. *JAMA Netw Open*. 2022;5(2):e2147053. doi:10.1001/jamanetworkopen.2021.47053

eTable 1. New symptom and condition ICD-10-CM diagnostic codes

eTable 2. Institutions contributing data

eTable 3. Prevalence of new diagnoses of symptoms and conditions among adults and children and young adults with positive or negative SARS-CoV-2 test results with medical encounters 31 to 150 days after first test, PCORnet, March to December 2020

This supplemental material has been provided by the authors to give readers additional information about their work.

© 2022 Hernandez-Romieu AC et al. *JAMA Network Open.*

eTable 1. New symptom and condition ICD-10-CM diagnostic codes.

**Non-specific Heart Rate Abnormalities**	
R00.0	Tachycardia, unspecified
R00.1	Bradycardia, unspecified
R00.2	Palpitations
**Change in Bowel Habits**	
K58	Irritable bowel syndrome
K58.0	Irritable bowel syndrome with diarrhea
K58.1	Irritable bowel syndrome with constipation
K58.2	Mixed irritable bowel syndrome
K58.8	Other irritable bowel syndrome
K58.9	Irritable bowel syndrome without diarrhea
K59	Constipation
K59.0	Constipation
K59.00	Constipation, unspecified
K59.01	Slow transit constipation
K59.02	Outlet dysfunction constipation
K59.03	Drug induced constipation
K59.04	Chronic idiopathic constipation
K59.09	Other constipation
K59.1	Functional diarrhea
K59.2	Neurogenic bowel, not elsewhere classified
K59.3	Megacolon, not elsewhere classified
K59.31	Toxic megacolon
K59.39	Other megacolon
K59.4	Anal spasm
K59.8	Other specified functional intestinal disorders
K59.81	Ogilvie syndrome
K59.89	Other specified functional intestinal disorders
K59.9	Functional intestinal disorder, unspecified
R19.4	Change in bowel habit
R19.8	Other specified symptoms and signs involving the digestive system and abdomen
**Autonomic Dysfunction**	
G90	Disorders of the autonomic nervous system
G90.0	Idiopathic peripheral autonomic neuropathy
G90.01	Carotid sinus syncope
G90.09	Other idiopathic peripheral autonomic neuropathy
G90.1	Familial dysautonomia [Riley-Day]
G90.2	Horner's syndrome
G90.3	Multi-system degeneration of the autonomic nervous system
G90.4	Autonomic dysreflexia
G90.5	Complex regional pain syndrome I (CRPS I)
G90.50	Complex regional pain syndrome I, unspecified
G90.51	Complex regional pain syndrome I of upper limb
G90.511	Complex regional pain syndrome I of right upper limb

© 2022 Hernandez-Romieu AC et al. *JAMA Network Open.*

G90.512	Complex regional pain syndrome I of left upper limb
G90.513	Complex regional pain syndrome I of upper limb, bilateral
G90.519	Complex regional pain syndrome I of unspecified upper limb
G90.52	Complex regional pain syndrome I of lower limb
G90.521	Complex regional pain syndrome I of right lower limb
G90.522	Complex regional pain syndrome I of left lower limb
G90.523	Complex regional pain syndrome I of lower limb, bilateral
G90.529	Complex regional pain syndrome I of unspecified lower limb
G90.59	Complex regional pain syndrome I of other specified site
G90.8	Other disorders of autonomic nervous system
G90.9	Disorder of the autonomic nervous system, unspecified
I49.8	Postural Orthostatic Tachycardia Syndrome
I95.1	Orthostatic hypotension
**Sensation and Perception**	
H53.10	Unspecified subjective visual disturbances
H53.14	Photophobia
H53.141	Visual discomfort, right eye
H53.142	Visual discomfort, left eye
H53.143	Visual discomfort, bilateral
H53.149	Visual discomfort, unspecified
H53.8	Other visual disturbances
H90.A	Hearing loss
H90.A1	Conductive hearing loss, unilateral, with restricted hearing on the contralateral side
H90.A11	Conductive hearing loss, unilateral, right ear with restricted hearing on the contralateral side
H90.A12	Conductive hearing loss, unilateral, left ear with restricted hearing on the contralateral side
H90.A2	Sensorineural hearing loss, unilateral, with restricted hearing on the contralateral side
H90.A21	Sensorineural hearing loss, unilateral, right ear, with restricted hearing on the contralateral side
H90.A22	Sensorineural hearing loss, unilateral, left ear, with restricted hearing on the contralateral side
H90.A3	Mixed conductive and sensorineural hearing loss, unilateral with restricted hearing on the contralateral side
H90.A31	Mixed conductive and sensorineural hearing loss, unilateral, right ear with restricted hearing on the contralateral side
H90.A32	Mixed conductive and sensorineural hearing loss, unilateral, left ear with restricted hearing on the contralateral side
H91.2	Other hearing loss
H91.20	Sudden idiopathic hearing loss, unspecified ear
H91.21	Sudden idiopathic hearing loss, right ear
H91.22	Sudden idiopathic hearing loss, left ear
H91.23	Sudden idiopathic hearing loss, bilateral
H91.9	Other hearing loss
H91.90	Unspecified hearing loss, unspecified ear
H91.91	Unspecified hearing loss, right ear
H91.92	Unspecified hearing loss, left ear
H91.93	Unspecified hearing loss, bilateral
H93.23	Hyperacusis

© 2022 Hernandez-Romieu AC et al. *JAMA Network Open.*

H93.231	Hyperacusis, right ear
H93.232	Hyperacusis, left ear
H93.233	Hyperacusis, bilateral
H93.239	Hyperacusis, unspecified ear
H93.29	Other abnormal auditory perceptions
H93.291	Other abnormal auditory perceptions, right ear
H93.292	Other abnormal auditory perceptions, left ear
H93.293	Other abnormal auditory perceptions, bilateral
H93.299	Other abnormal auditory perceptions, unspecified ear
R44.0	Auditory hallucinations
R44.1	Visual hallucinations
R44.2	Other hallucinations
R44.8	Other symptoms and signs involving general sensations and perceptions
R44.9	Unspecified symptoms and signs involving general sensations and perceptions
**Anxiety and Depression**	
F06.31	Mood disorder due to known physiol cond w depressv features
F06.32	Mood disord d/t physiol cond w major depressive-like epsd
F06.34	Mood disorder due to known physiological condition with mixed features
F32.0	Major depressive disorder, single episode, mild
F32.1	Major depressive disorder, single episode, moderate
F32.2	Major depressv disord, single epsd, sev w/o psych features
F32.3	Major depressv disord, single epsd, severe w psych features
F32.4	Major depressv disorder, single episode, in partial remis
F32.5	Major depressive disorder, single episode, in full remission
F32.8	Other depressive episodes
F32.89	Other specified depressive episodes
F32.9	Major depressive disorder, single episode, unspecified
F33.0	Major depressive disorder, recurrent, mild
F33.1	Major depressive disorder, recurrent, moderate
F33.2	Major depressv disorder, recurrent severe w/o psych features
F33.3	Major depressv disorder, recurrent, severe w psych symptoms
F33.40	Major depressive disorder, recurrent, in remission, unsp
F33.41	Major depressive disorder, recurrent, in partial remission
F33.42	Major depressive disorder, recurrent, in full remission
F33.8	Other recurrent depressive disorders
F33.9	Major depressive disorder, recurrent, unspecified
F34.0	Cyclothymic disorder
F34.1	Dysthymic disorder
F34.8	Other persistent mood [affective] disorders
F34.81	Disruptive mood dysregulation disorder
F34.89	Other specified persistent mood disorders
F34.9	Persistent mood [affective] disorder, unspecified
F39	Unspecified mood [affective] disorder
F41.1	Generalized anxiety disorder
F45.0	Nervousness

© 2022 Hernandez-Romieu AC et al. *JAMA Network Open.*

R45.1	Restlessness and agitation
R45.2	Unhappiness
R45.4	Demoralization and apathy
R45.7	State of emotional shock and stress, unspecified
R45.84	Anhedonia
**Fatigue**	
G93.3	Post-viral fatigue syndrome (Myalgic encephalomyelitis)
R40.0	Somnolence
R53	Malaise and fatigue
R53.1	Weakness
R53.8	Other malaise and fatigue
R53.81	Other malaise
R53.82	Chronic fatigue, unspecified
R53.83	Other fatigue
**Sleep Disorders**	
F51	Sleep disorder not from a physiologic condition
F51.0	Insomnia not due to a substance or known physiological condition
F51.01	Primary insomnia
F51.02	Adjustment insomnia
F51.03	Paradoxical insomnia
F51.04	Psychophysiologic insomnia
F51.05	Insomnia due to other mental disorder
F51.09	Other insomnia not due to a substance or known physiological condition
F51.1	Hypersomnia not due to a substance or known physiological condition
F51.11	Primary hypersomnia
F51.12	Insufficient sleep syndrome
F51.13	Hypersomnia due to other mental disorder
F51.19	Other hypersomnia not due to a substance or known physiological condition
F51.3	Sleepwalking [somnambulism]
F51.4	Sleep terrors [night terrors]
F51.5	Nightmare disorder
F51.8	Other sleep disorders not due to a substance or known physiological condition
F51.9	Sleep disorder not due to a substance or known physiological condition, unspecified
G47	Sleep disorders such as insomnia and hyperosmia
G47.0	Insomnia
G47.00	Insomnia, unspecified
G47.01	Insomnia due to medical condition
G47.09	Other insomnia
G47.1	Hypersomnia
G47.10	Hypersomnia, unspecified
G47.11	Idiopathic hypersomnia with long sleep time
G47.12	Idiopathic hypersomnia without long sleep time
G47.13	Recurrent hypersomnia
G47.14	Hypersomnia due to medical condition
G47.19	Other hypersomnia

© 2022 Hernandez-Romieu AC et al. *JAMA Network Open.*

G47.2	Circadian rhythm sleep disorders
G47.20	Circadian rhythm sleep disorder, unspecified type
G47.21	Circadian rhythm sleep disorder, delayed sleep phase type
G47.22	Circadian rhythm sleep disorder, advanced sleep phase type
G47.23	Circadian rhythm sleep disorder, irregular sleep wake type
G47.24	Circadian rhythm sleep disorder, free running type
G47.25	Circadian rhythm sleep disorder, jet lag type
G47.26	Circadian rhythm sleep disorder, shift work type
G47.27	Circadian rhythm sleep disorder in conditions classified elsewhere
G47.29	Other circadian rhythm sleep disorder
G47.3	Sleep apnea
G47.30	Sleep apnea, unspecified
G47.31	Primary central sleep apnea
G47.32	High altitude periodic breathing
G47.33	Obstructive sleep apnea (adult) (pediatric)
G47.34	Idiopathic sleep related nonobstructive alveolar hypoventilation
G47.35	Congenital central alveolar hypoventilation syndrome
G47.36	Sleep related hypoventilation in conditions classified elsewhere
G47.37	Central sleep apnea in conditions classified elsewhere
G47.39	Other sleep apnea
G47.4	Narcolepsy and cataplexy
G47.41	Narcolepsy
G47.411	Narcolepsy with cataplexy
G47.419	Narcolepsy without cataplexy
G47.42	Narcolepsy in conditions classified elsewhere
G47.421	Narcolepsy in conditions classified elsewhere with cataplexy
G47.429	Narcolepsy in conditions classified elsewhere without cataplexy
G47.5	Parasomnia
G47.50	Parasomnia, unspecified
G47.51	Confusional arousals
G47.52	REM sleep behavior disorder
G47.53	Recurrent isolated sleep paralysis
G47.54	Parasomnia in conditions classified elsewhere
G47.59	Other parasomnia
G47.6	Sleep related movement disorders
G47.61	Periodic limb movement disorder
G47.62	Sleep related leg cramps
G47.63	Sleep related bruxism
G47.69	Other sleep related movement disorders
G47.8	Other sleep disorders
G47.9	Sleep disorder, unspecified
**Headache**	
G43	Migraine
G43.0	Migraine without aura
G43.00	Migraine without aura, not intractable

© 2022 Hernandez-Romieu AC et al. *JAMA Network Open.*

G43.001	Migraine without aura, not intractable, with status migrainosus
G43.009	Migraine without aura, not intractable, without status migrainosus
G43.01	Migraine without aura, intractable
G43.011	Migraine without aura, intractable, with status migrainosus
G43.019	Migraine without aura, intractable, without status migrainosus
G43.1	Migraine with aura
G43.10	Migraine with aura, not intractable
G43.101	Migraine with aura, not intractable, with status migrainosus
G43.109	Migraine with aura, not intractable, without status migrainosus
G43.11	Migraine with aura, intractable
G43.111	Migraine with aura, intractable, with status migrainosus
G43.119	Migraine with aura, intractable, without status migrainosus
G43.4	Hemiplegic migraine
G43.40	Hemiplegic migraine, not intractable
G43.401	Hemiplegic migraine, not intractable, with status migrainosus
G43.409	Hemiplegic migraine, not intractable, without status migrainosus
G43.41	Hemiplegic migraine, intractable
G43.411	Hemiplegic migraine, intractable, with status migrainosus
G43.419	Hemiplegic migraine, intractable, without status migrainosus
G43.5	Persistent migraine aura without cerebral infarction
G43.50	Persistent migraine aura without cerebral infarction, not intractable
G43.501	Persistent migraine aura without cerebral infarction, not intractable, with status migrainosus
G43.509	Persistent migraine aura without cerebral infarction, not intractable, without status migrainosus
G43.51	Persistent migraine aura without cerebral infarction, intractable
G43.511	Persistent migraine aura without cerebral infarction, intractable, with status migrainosus
G43.519	Persistent migraine aura without cerebral infarction, intractable, without status migrainosus
G43.6	Persistent migraine aura with cerebral infarction
G43.60	Persistent migraine aura with cerebral infarction, not intractable
G43.601	Persistent migraine aura with cerebral infarction, not intractable, with status migrainosus
G43.609	Persistent migraine aura with cerebral infarction, not intractable, without status migrainosus
G43.61	Persistent migraine aura with cerebral infarction, intractable
G43.611	Persistent migraine aura with cerebral infarction, intractable, with status migrainosus
G43.619	Persistent migraine aura with cerebral infarction, intractable, without status migrainosus
G43.7	Chronic migraine without aura
G43.70	Chronic migraine without aura, not intractable
G43.701	Chronic migraine without aura, not intractable, with status migrainosus
G43.709	Chronic migraine without aura, not intractable, without status migrainosus
G43.71	Chronic migraine without aura, intractable
G43.711	Chronic migraine without aura, intractable, with status migrainosus
G43.719	Chronic migraine without aura, intractable, without status migrainosus
G43.8	Other migraine
G43.80	Other migraine, not intractable
G43.801	Other migraine, not intractable, with status migrainosus
G43.809	Other migraine, not intractable, without status migrainosus
G43.81	Other migraine, intractable

© 2022 Hernandez-Romieu AC et al. *JAMA Network Open.*

G43.811	Other migraine, intractable, with status migrainosus
G43.819	Other migraine, intractable, without status migrainosus
G43.82	Menstrual migraine, not intractable
G43.821	Menstrual migraine, not intractable, with status migrainosus
G43.829	Menstrual migraine, not intractable, without status migrainosus
G43.83	Menstrual migraine, intractable
G43.831	Menstrual migraine, intractable, with status migrainosus
G43.839	Menstrual migraine, intractable, without status migrainosus
G43.9	Migraine, unspecified
G43.90	Migraine, unspecified, not intractable
G43.901	Migraine, unspecified, not intractable, with status migrainosus
G43.909	Migraine, unspecified, not intractable, without status migrainosus
G43.91	Migraine, unspecified, intractable
G43.911	Migraine, unspecified, intractable, with status migrainosus
G43.919	Migraine, unspecified, intractable, without status migrainosus
G43.A	Cyclical vomiting
G43.A0	Cyclical vomiting, in migraine, not intractable
G43.A1	Cyclical vomiting, in migraine, intractable
G43.B	Ophthalmoplegic migraine
G43.B0	Ophthalmoplegic migraine, not intractable
G43.B1	Ophthalmoplegic migraine, intractable
G43.C	Periodic headache syndromes in child or adult
G43.C0	Periodic headache syndromes in child or adult, not intractable
G43.C1	Periodic headache syndromes in child or adult, intractable
G43.D	Abdominal migraine
G43.D0	Abdominal migraine, not intractable
G43.D1	Abdominal migraine, intractable
G44	Other headache syndromes
G44.0	Cluster headache and other trigeminal autonomic cephalgias
G44.00	Cluster headache syndrome, unspecified
G44.001	Cluster headache syndrome, unspecified, intractable
G44.009	Cluster headache syndrome, unspecified, not intractable
G44.01	Episodic cluster headache
G44.011	Episodic cluster headache, intractable
G44.019	Episodic cluster headache, not intractable
G44.02	Chronic cluster headache
G44.021	Chronic cluster headache, intractable
G44.029	Chronic cluster headache, not intractable
G44.03	Episodic paroxysmal hemicrania
G44.031	Episodic paroxysmal hemicrania, intractable
G44.039	Episodic paroxysmal hemicrania, not intractable
G44.04	Chronic paroxysmal hemicrania
G44.041	Chronic paroxysmal hemicrania, intractable
G44.049	Chronic paroxysmal hemicrania, not intractable
G44.05	Short lasting unilateral neuralgiform headache with conjunctival injection and tearing (SUNCT)

© 2022 Hernandez-Romieu AC et al. *JAMA Network Open.*

G44.051	Short lasting unilateral neuralgiform headache with conjunctival injection and tearing (SUNCT), intractable
G44.059	Short lasting unilateral neuralgiform headache with conjunctival injection and tearing (SUNCT), not intractable
G44.09	Other trigeminal autonomic cephalgias (TAC)
G44.091	Other trigeminal autonomic cephalgias (TAC), intractable
G44.099	Other trigeminal autonomic cephalgias (TAC), not intractable
G44.1	Vascular headache, not elsewhere specified
G44.2	Tension-type headache
G44.20	Tension-type headache, unspecified
G44.201	Tension-type headache, unspecified, intractable
G44.209	Tension-type headache, unspecified, not intractable
G44.21	Episodic tension-type headache
G44.211	Episodic tension-type headache, intractable
G44.219	Episodic tension-type headache, not intractable
G44.22	Chronic tension-type headache
G44.221	Chronic tension-type headache, intractable
G44.229	Chronic tension-type headache, not intractable
G44.3	Post-traumatic headache
G44.30	Post-traumatic headache, unspecified
G44.301	Post-traumatic headache, unspecified, intractable
G44.309	Post-traumatic headache, unspecified, not intractable
G44.31	Acute post-traumatic headache
G44.311	Acute post-traumatic headache, intractable
G44.319	Acute post-traumatic headache, not intractable
G44.32	Chronic post-traumatic headache
G44.321	Chronic post-traumatic headache, intractable
G44.329	Chronic post-traumatic headache, not intractable
G44.4	Drug-induced headache, not elsewhere classified
G44.40	Drug-induced headache, not elsewhere classified, not intractable
G44.41	Drug-induced headache, not elsewhere classified, intractable
G44.5	Complicated headache syndromes
G44.51	Hemicrania continua
G44.52	New daily persistent headache (NDPH)
G44.53	Primary thunderclap headache
G44.59	Other complicated headache syndrome
G44.8	Other specified headache syndromes
G44.81	Hypnic headache
G44.82	Headache associated with sexual activity
G44.83	Primary cough headache
G44.84	Primary exertional headache
G44.85	Primary stabbing headache
G44.89	Other headache syndrome
R51.0	Headache with orthostatic component
R51.9	Headache, unspecified
**Myoneural Disorders**	

© 2022 Hernandez-Romieu AC et al. *JAMA Network Open.*

G70	Myasthenia gravis and other myoneural disorders
G70.0	Myasthenia gravis
G70.00	Myasthenia gravis without (acute) exacerbation
G70.01	Myasthenia gravis with (acute) exacerbation
G70.1	Toxic myoneural disorders
G70.2	Congenital and developmental myasthenia
G70.8	Other specified myoneural disorders
G70.80	Lambert-Eaton syndrome
G70.81	Lambert-Eaton syndrome in disease classified elsewhere
G70.89	Other specified myoneural disorders
G70.9	Myoneural disorder, unspecified
G71.9	Primary disorders of muscle, unspecified
G72.81	Critical illness myopathy
G72.89	Other specified myopathies
G72.9	Myopathy, unspecified
M60	Myositis
M60.0	Infective myositis
M60.00	Infective myositis, unspecified site
M60.000	Infective myositis, unspecified right arm
M60.001	Infective myositis, unspecified left arm
M60.002	Infective myositis, unspecified arm
M60.003	Infective myositis, unspecified right leg
M60.004	Infective myositis, unspecified left leg
M60.005	Infective myositis, unspecified leg
M60.009	Infective myositis, unspecified site
M60.01	Infective myositis, shoulder
M60.011	Infective myositis, right shoulder
M60.012	Infective myositis, left shoulder
M60.019	Infective myositis, unspecified shoulder
M60.02	Infective myositis, upper arm
M60.021	Infective myositis, right upper arm
M60.022	Infective myositis, left upper arm
M60.029	Infective myositis, unspecified upper arm
M60.03	Infective myositis, forearm
M60.031	Infective myositis, right forearm
M60.032	Infective myositis, left forearm
M60.039	Infective myositis, unspecified forearm
M60.04	Infective myositis, hand and fingers
M60.041	Infective myositis, right hand
M60.042	Infective myositis, left hand
M60.043	Infective myositis, unspecified hand
M60.044	Infective myositis, right finger(s)
M60.045	Infective myositis, left finger(s)
M60.046	Infective myositis, unspecified finger(s)
M60.05	Infective myositis, thigh

© 2022 Hernandez-Romieu AC et al. *JAMA Network Open.*

M60.051	Infective myositis, right thigh
M60.052	Infective myositis, left thigh
M60.059	Infective myositis, unspecified thigh
M60.06	Infective myositis, lower leg
M60.061	Infective myositis, right lower leg
M60.062	Infective myositis, left lower leg
M60.069	Infective myositis, unspecified lower leg
M60.07	Infective myositis, ankle, foot and toes
M60.070	Infective myositis, right ankle
M60.071	Infective myositis, left ankle
M60.072	Infective myositis, unspecified ankle
M60.073	Infective myositis, right foot
M60.074	Infective myositis, left foot
M60.075	Infective myositis, unspecified foot
M60.076	Infective myositis, right toe(s)
M60.077	Infective myositis, left toe(s)
M60.078	Infective myositis, unspecified toe(s)
M60.08	Infective myositis, other site
M60.09	Infective myositis, multiple sites
M60.1	Interstitial myositis
M60.10	Interstitial myositis of unspecified site
M60.11	Interstitial myositis, shoulder
M60.111	Interstitial myositis, right shoulder
M60.112	Interstitial myositis, left shoulder
M60.119	Interstitial myositis, unspecified shoulder
M60.12	Interstitial myositis, upper arm
M60.121	Interstitial myositis, right upper arm
M60.122	Interstitial myositis, left upper arm
M60.129	Interstitial myositis, unspecified upper arm
M60.13	Interstitial myositis, forearm
M60.131	Interstitial myositis, right forearm
M60.132	Interstitial myositis, left forearm
M60.139	Interstitial myositis, unspecified forearm
M60.14	Interstitial myositis, hand
M60.141	Interstitial myositis, right hand
M60.142	Interstitial myositis, left hand
M60.149	Interstitial myositis, unspecified hand
M60.15	Interstitial myositis, thigh
M60.151	Interstitial myositis, right thigh
M60.152	Interstitial myositis, left thigh
M60.159	Interstitial myositis, unspecified thigh
M60.16	Interstitial myositis, lower leg
M60.161	Interstitial myositis, right lower leg
M60.162	Interstitial myositis, left lower leg
M60.169	Interstitial myositis, unspecified lower leg

© 2022 Hernandez-Romieu AC et al. *JAMA Network Open.*

M60.17	Interstitial myositis, ankle and foot
M60.171	Interstitial myositis, right ankle and foot
M60.172	Interstitial myositis, left ankle and foot
M60.179	Interstitial myositis, unspecified ankle and foot
M60.18	Interstitial myositis, other site
M60.19	Interstitial myositis, multiple sites
M60.2	Foreign body granuloma of soft tissue, not elsewhere classified
M60.20	Foreign body granuloma of soft tissue, not elsewhere classified, unspecified site
M60.21	Foreign body granuloma of soft tissue, not elsewhere classified, shoulder
M60.211	Foreign body granuloma of soft tissue, not elsewhere classified, right shoulder
M60.212	Foreign body granuloma of soft tissue, not elsewhere classified, left shoulder
M60.219	Foreign body granuloma of soft tissue, not elsewhere classified, unspecified shoulder
M60.22	Foreign body granuloma of soft tissue, not elsewhere classified, upper arm
M60.221	Foreign body granuloma of soft tissue, not elsewhere classified, right upper arm
M60.222	Foreign body granuloma of soft tissue, not elsewhere classified, left upper arm
M60.229	Foreign body granuloma of soft tissue, not elsewhere classified, unspecified upper arm
M60.23	Foreign body granuloma of soft tissue, not elsewhere classified, forearm
M60.231	Foreign body granuloma of soft tissue, not elsewhere classified, right forearm
M60.232	Foreign body granuloma of soft tissue, not elsewhere classified, left forearm
M60.239	Foreign body granuloma of soft tissue, not elsewhere classified, unspecified forearm
M60.24	Foreign body granuloma of soft tissue, not elsewhere classified, hand
M60.241	Foreign body granuloma of soft tissue, not elsewhere classified, right hand
M60.242	Foreign body granuloma of soft tissue, not elsewhere classified, left hand
M60.249	Foreign body granuloma of soft tissue, not elsewhere classified, unspecified hand
M60.25	Foreign body granuloma of soft tissue, not elsewhere classified, thigh
M60.251	Foreign body granuloma of soft tissue, not elsewhere classified, right thigh
M60.252	Foreign body granuloma of soft tissue, not elsewhere classified, left thigh
M60.259	Foreign body granuloma of soft tissue, not elsewhere classified, unspecified thigh
M60.26	Foreign body granuloma of soft tissue, not elsewhere classified, lower leg
M60.261	Foreign body granuloma of soft tissue, not elsewhere classified, right lower leg
M60.262	Foreign body granuloma of soft tissue, not elsewhere classified, left lower leg
M60.269	Foreign body granuloma of soft tissue, not elsewhere classified, unspecified lower leg
M60.27	Foreign body granuloma of soft tissue, not elsewhere classified, ankle and foot
M60.271	Foreign body granuloma of soft tissue, not elsewhere classified, right ankle and foot
M60.272	Foreign body granuloma of soft tissue, not elsewhere classified, left ankle and foot
M60.279	Foreign body granuloma of soft tissue, not elsewhere classified, unspecified ankle and foot
M60.28	Foreign body granuloma of soft tissue, not elsewhere classified, other site
M60.8	Other myositis
M60.80	Other myositis, unspecified site
M60.81	Other myositis shoulder
M60.811	Other myositis, right shoulder
M60.812	Other myositis, left shoulder
M60.819	Other myositis, unspecified shoulder
M60.82	Other myositis, upper arm
M60.821	Other myositis, right upper arm

M60.822	Other myositis, left upper arm
M60.829	Other myositis, unspecified upper arm
M60.83	Other myositis, forearm
M60.831	Other myositis, right forearm
M60.832	Other myositis, left forearm
M60.839	Other myositis, unspecified forearm
M60.84	Other myositis, hand
M60.841	Other myositis, right hand
M60.842	Other myositis, left hand
M60.849	Other myositis, unspecified hand
M60.85	Other myositis, thigh
M60.851	Other myositis, right thigh
M60.852	Other myositis, left thigh
M60.859	Other myositis, unspecified thigh
M60.86	Other myositis, lower leg
M60.861	Other myositis, right lower leg
M60.862	Other myositis, left lower leg
M60.869	Other myositis, unspecified lower leg
M60.87	Other myositis, ankle and foot
M60.871	Other myositis, right ankle and foot
M60.872	Other myositis, left ankle and foot
M60.879	Other myositis, unspecified ankle and foot
M60.88	Other myositis, other site
M60.89	Other myositis, multiple sites
M60.9	Myositis, unspecified
**Symptoms and signs involving cognition, perception, emotional state and behavior**	
R40.1	Stupor, semicoma
R40.2	Unspecified coma
R40.20	Unspecified coma
R40.21	Coma scale, eyes open
R40.211	Coma scale, eyes open, never
R40.2110	Coma scale, eyes open, never, unspecified time
R40.2111	Coma scale, eyes open, never, in the field [EMT or ambulance]
R40.2112	Coma scale, eyes open, never, at arrival to emergency department
R40.2113	Coma scale, eyes open, never, at hospital admission
R40.2114	Coma scale, eyes open, never, 24 hours or more after hospital admission
R40.212	Coma scale, eyes open, to pain
R40.2120	Coma scale, eyes open, to pain, unspecified time
R40.2121	Coma scale, eyes open, to pain, in the field [EMT or ambulance]
R40.2122	Coma scale, eyes open, to pain, at arrival to emergency department
R40.2123	Coma scale, eyes open, to pain, at hospital admission
R40.2124	Coma scale, eyes open, to pain, 24 hours or more after hospital admission
R40.213	Coma scale, eyes open, to sound
R40.2130	Coma scale, eyes open, to sound, unspecified time
R40.2131	Coma scale, eyes open, to sound, in the field [EMT or ambulance]

© 2022 Hernandez-Romieu AC et al. *JAMA Network Open.*

R40.2132	Coma scale, eyes open, to sound, at arrival to emergency department
R40.2133	Coma scale, eyes open, to sound, at hospital admission
R40.2134	Coma scale, eyes open, to sound, 24 hours or more after hospital admission
R40.214	Coma scale, eyes open, spontaneous
R40.2140	Coma scale, eyes open, spontaneous, unspecified time
R40.2141	Coma scale, eyes open, spontaneous, in the field [EMT or ambulance]
R40.2142	Coma scale, eyes open, spontaneous, at arrival to emergency department
R40.2143	Coma scale, eyes open, spontaneous, at hospital admission
R40.2144	Coma scale, eyes open, spontaneous, 24 hours or more after hospital admission
R40.22	Coma scale, best verbal response
R40.221	Coma scale, best verbal response, none
R40.2210	Coma scale, best verbal response, none, unspecified time
R40.2211	Coma scale, best verbal response, none, in the field [EMT or ambulance]
R40.2212	Coma scale, best verbal response, none, at arrival to emergency department
R40.2213	Coma scale, best verbal response, none, at hospital admission
R40.2214	Coma scale, best verbal response, none, 24 hours or more after hospital admission
R40.222	Coma scale, best verbal response, incomprehensible words
R40.2220	Coma scale, best verbal response, incomprehensible words, unspecified time
R40.2221	Coma scale, best verbal response, incomprehensible words, in the field [EMT or ambulance]
R40.2222	Coma scale, best verbal response, incomprehensible words, at arrival to emergency department
R40.2223	Coma scale, best verbal response, incomprehensible words, at hospital admission
R40.2224	Coma scale, best verbal response, incomprehensible words, 24 hours or more after hospital admission
R40.223	Coma scale, best verbal response, inappropriate words
R40.2230	Coma scale, best verbal response, inappropriate words, unspecified time
R40.2231	Coma scale, best verbal response, inappropriate words, in the field [EMT or ambulance]
R40.2232	Coma scale, best verbal response, inappropriate words, at arrival to emergency department
R40.2233	Coma scale, best verbal response, inappropriate words, at hospital admission
R40.2234	Coma scale, best verbal response, inappropriate words, 24 hours or more after hospital admission
R40.224	Coma scale, best verbal response, confused conversation
R40.2240	Coma scale, best verbal response, confused conversation, unspecified time
R40.2241	Coma scale, best verbal response, confused conversation, in the field [EMT or ambulance]
R40.2242	Coma scale, best verbal response, confused conversation, at arrival to emergency department
R40.2243	Coma scale, best verbal response, confused conversation, at hospital admission
R40.2244	Coma scale, best verbal response, confused conversation, 24 hours or more after hospital admission
R40.225	Coma scale, best verbal response, oriented
R40.2250	Coma scale, best verbal response, oriented, unspecified time
R40.2251	Coma scale, best verbal response, oriented, in the field [EMT or ambulance]
R40.2252	Coma scale, best verbal response, oriented, at arrival to emergency department
R40.2253	Coma scale, best verbal response, oriented, at hospital admission
R40.2254	Coma scale, best verbal response, oriented, 24 hours or more after hospital admission
R40.23	Coma scale, best motor response
R40.231	Coma scale, best motor response, none

R40.2310	Coma scale, best motor response, none, unspecified time
R40.2311	Coma scale, best motor response, none, in the field [EMT or ambulance]
R40.2312	Coma scale, best motor response, none, at arrival to emergency department
R40.2313	Coma scale, best motor response, none, at hospital admission
R40.2314	Coma scale, best motor response, none, 24 hours or more after hospital admission
R40.232	Coma scale, best motor response, extension
R40.2320	Coma scale, best motor response, extension, unspecified time
R40.2321	Coma scale, best motor response, extension, in the field [EMT or ambulance]
R40.2322	Coma scale, best motor response, extension, at arrival to emergency department
R40.2323	Coma scale, best motor response, extension, at hospital admission
R40.2324	Coma scale, best motor response, extension, 24 hours or more after hospital admission
R40.233	Coma scale, best motor response, abnormal flexion
R40.2330	Coma scale, best motor response, abnormal flexion, unspecified time
R40.2331	Coma scale, best motor response, abnormal flexion, in the field [EMT or ambulance]
R40.2332	Coma scale, best motor response, abnormal flexion, at arrival to emergency department
R40.2333	Coma scale, best motor response, abnormal flexion, at hospital admission
R40.2334	Coma scale, best motor response, abnormal flexion, 24 hours or more after hospital admission
R40.234	Coma scale, best motor response, flexion withdrawal
R40.2340	Coma scale, best motor response, flexion withdrawal, unspecified time
R40.2341	Coma scale, best motor response, flexion withdrawal, in the field [EMT or ambulance]
R40.2342	Coma scale, best motor response, flexion withdrawal, at arrival to emergency department
R40.2343	Coma scale, best motor response, flexion withdrawal, at hospital admission
R40.2344	Coma scale, best motor response, flexion withdrawal, 24 hours or more after hospital admission
R40.235	Coma scale, best motor response, localizes pain
R40.2350	Coma scale, best motor response, localizes pain, unspecified time
R40.2351	Coma scale, best motor response, localizes pain, in the field [EMT or ambulance]
R40.2352	Coma scale, best motor response, localizes pain, at arrival to emergency department
R40.2353	Coma scale, best motor response, localizes pain, at hospital admission
R40.2354	Coma scale, best motor response, localizes pain, 24 hours or more after hospital admission
R40.236	Coma scale, best motor response, obeys commands
R40.2360	Coma scale, best motor response, obeys commands, unspecified time
R40.2361	Coma scale, best motor response, obeys commands, in the field [EMT or ambulance]
R40.2362	Coma scale, best motor response, obeys commands, at arrival to emergency department
R40.2363	Coma scale, best motor response, obeys commands, at hospital admission
R40.2364	Coma scale, best motor response, obeys commands, 24 hours or more after hospital admission
R40.24	Glasgow coma scale, total score
R40.241	Glasgow coma scale score 13-15
R40.2410	Glasgow coma scale score 13-15, unspecified time
R40.2411	Glasgow coma scale score 13-15, in the field [EMT or ambulance]
R40.2412	Glasgow coma scale score 13-15, at arrival to emergency department
R40.2413	Glasgow coma scale score 13-15, at hospital admission
R40.2414	Glasgow coma scale score 13-15, 24 hours or more after hospital admission
R40.242	Glasgow coma scale score 9-12
R40.2420	Glasgow coma scale score 9-12, unspecified time

© 2022 Hernandez-Romieu AC et al. *JAMA Network Open.*

R40.2421	Glasgow coma scale score 9-12, in the field [EMT or ambulance]
R40.2422	Glasgow coma scale score 9-12, at arrival to emergency department
R40.2423	Glasgow coma scale score 9-12, at hospital admission
R40.2424	Glasgow coma scale score 9-12, 24 hours or more after hospital admission
R40.243	Glasgow coma scale score 3-8
R40.2430	Glasgow coma scale score 3-8, unspecified time
R40.2431	Glasgow coma scale score 3-8, in the field [EMT or ambulance]
R40.2432	Glasgow coma scale score 3-8, at arrival to emergency department
R40.2433	Glasgow coma scale score 3-8, at hospital admission
R40.2434	Glasgow coma scale score 3-8, 24 hours or more after hospital admission
R40.244	Other coma, without documented Glasgow coma scale score, or with partial score reported
R40.2440	Other coma, without documented Glasgow coma scale score, or with partial score reported, unspecified time
R40.2441	Other coma, without documented Glasgow coma scale score, or with partial score reported, in the field [EMT or ambulance]
R40.2442	Other coma, without documented Glasgow coma scale score, or with partial score reported, at arrival to emergency department
R40.2443	Other coma, without documented Glasgow coma scale score, or with partial score reported, at hospital admission
R40.2444	Other coma, without documented Glasgow coma scale score, or with partial score reported, 24 hours or more after hospital admission
R41.0	Disorientation, unspecified, confusion
R41.1	Anterograde amnesia
R41.2	Retrograde amnesia
R41.3	Other amnesia
R41.4	Other and unspecified symptoms and signs involving cognitive functions and awareness
R41.8	Other and unspecified symptoms and signs involving cognitive functions and awareness
R41.81	Age-related cognitive decline
R41.82	Altered mental status, unspecified
R41.83	Borderline intellectual functioning
R41.84	Other specified cognitive deficit
R41.840	Attention and concentration deficit
R41.841	Cognitive communication deficit
R41.842	Visuospatial deficit
R41.843	Psychomotor deficit
R41.844	Frontal lobe and executive function deficit
R41.89	Other symptoms and signs involving cognitive functions and awareness
R42	Dizziness and giddiness (including light headedness and Vertigo)
R46.4	Slowness or poor responsiveness
**Shortness of Breath**	
R06.0	Dyspnea
R06.00	Dyspnea, unspecified
R06.02	Shortness of breath
R06.09	Other forms of dyspnea
R06.2	Wheezing
R06.82	Tachypnea, not elsewhere classified

© 2022 Hernandez-Romieu AC et al. *JAMA Network Open.*

R06.89	Other abnormalities of breathing
R06.9	Unspecified abnormalities of breathing
**Weight Loss**	
R63.4	Abnormal weight loss
R64	Cachexia
260*	WTLOSS_ELIX
261*	WTLOSS_ELIX
262*	WTLOSS_ELIX
263*	WTLOSS_ELIX
E40	WTLOSS_ELIX
E41	WTLOSS_ELIX
E42	WTLOSS_ELIX
E43	WTLOSS_ELIX
E44	WTLOSS_ELIX
E440	WTLOSS_ELIX
E441	WTLOSS_ELIX
E45	WTLOSS_ELIX
E46	WTLOSS_ELIX
E640	WTLOSS_ELIX
**Peripheral Nerve Disorders**	
G50	Disorders of trigeminal nerve
G50.0	Trigeminal neuralgia
G50.1	Atypical facial pain
G50.8	Other disorders of trigeminal nerve
G50.9	Disorder of trigeminal nerve, unspecified
G51	Facial nerve disorders
G51.0	Bell's palsy
G51.1	Geniculate ganglionitis
G51.2	Melkersson's syndrome
G51.3	Clonic hemifacial spasm
G51.31	Clonic hemifacial spasm, right
G51.32	Clonic hemifacial spasm, left
G51.33	Clonic hemifacial spasm, bilateral
G51.39	Clonic hemifacial spasm, unspecified
G51.4	Facial myokymia
G51.8	Other disorders of facial nerve
G51.9	Disorder of facial nerve, unspecified
G52	Disorders of other cranial nerves
G52.0	Disorders of olfactory nerve
G52.1	Disorders of glossopharyngeal nerve
G52.2	Disorders of vagus nerve
G52.3	Disorders of hypoglossal nerve
G52.7	Disorders of multiple cranial nerves
G52.8	Disorders of other specified cranial nerves
G52.9	Cranial nerve disorder, unspecified

© 2022 Hernandez-Romieu AC et al. *JAMA Network Open.*

G53	Cranial nerve disorders in diseases classified elsewhere
G54	Nerve root and plexus disorders
G54.0	Brachial plexus disorders
G54.1	Lumbosacral plexus disorders
G54.2	Cervical root disorders, not elsewhere classified
G54.3	Thoracic root disorders, not elsewhere classified
G54.4	Lumbosacral root disorders, not elsewhere classified
G54.5	Neuralgic amyotrophy
G54.6	Phantom limb syndrome with pain
G54.7	Phantom limb syndrome without pain
G54.8	Other nerve root and plexus disorders
G54.9	Nerve root and plexus disorder, unspecified
G56	Mononeuropathies of upper limb
G56.0	Carpal tunnel syndrome
G56.00	Carpal tunnel syndrome, unspecified upper limb
G56.01	Carpal tunnel syndrome, right upper limb
G56.02	Carpal tunnel syndrome, left upper limb
G56.03	Carpal tunnel syndrome, bilateral upper limbs
G56.1	Other lesions of median nerve
G56.10	Other lesions of median nerve, unspecified upper limb
G56.11	Other lesions of median nerve, right upper limb
G56.12	Other lesions of median nerve, left upper limb
G56.13	Other lesions of median nerve, bilateral upper limbs
G56.2	Lesion of ulnar nerve
G56.20	Lesion of ulnar nerve, unspecified upper limb
G56.21	Lesion of ulnar nerve, right upper limb
G56.22	Lesion of ulnar nerve, left upper limb
G56.23	Lesion of ulnar nerve, bilateral upper limbs
G56.3	Lesion of radial nerve
G56.30	Lesion of radial nerve, unspecified upper limb
G56.31	Lesion of radial nerve, right upper limb
G56.32	Lesion of radial nerve, left upper limb
G56.33	Lesion of radial nerve, bilateral upper limbs
G56.4	Causalgia of upper limb
G56.40	Causalgia of unspecified upper limb
G56.41	Causalgia of right upper limb
G56.42	Causalgia of left upper limb
G56.43	Causalgia of bilateral upper limbs
G56.8	Other specified mononeuropathies of upper limb
G56.80	Other specified mononeuropathies of unspecified upper limb
G56.81	Other specified mononeuropathies of right upper limb
G56.82	Other specified mononeuropathies of left upper limb
G56.83	Other specified mononeuropathies of bilateral upper limbs
G56.9	Unspecified mononeuropathy of upper limb
G56.90	Unspecified mononeuropathy of unspecified upper limb

© 2022 Hernandez-Romieu AC et al. *JAMA Network Open.*

G56.91	Unspecified mononeuropathy of right upper limb
G56.92	Unspecified mononeuropathy of left upper limb
G56.93	Unspecified mononeuropathy of bilateral upper limbs
G57	Mononeuropathies of lower limb
G57.0	Lesion of sciatic nerve
G57.00	Lesion of sciatic nerve, unspecified lower limb
G57.01	Lesion of sciatic nerve, right lower limb
G57.02	Lesion of sciatic nerve, left lower limb
G57.03	Lesion of sciatic nerve, bilateral lower limbs
G57.1	Meralgia paresthetica
G57.10	Meralgia paresthetica, unspecified lower limb
G57.11	Meralgia paresthetica, right lower limb
G57.12	Meralgia paresthetica, left lower limb
G57.13	Meralgia paresthetica, bilateral lower limbs
G57.2	Lesion of femoral nerve
G57.20	Lesion of femoral nerve, unspecified lower limb
G57.21	Lesion of femoral nerve, right lower limb
G57.22	Lesion of femoral nerve, left lower limb
G57.23	Lesion of femoral nerve, bilateral lower limbs
G57.3	Lesion of lateral popliteal nerve
G57.30	Lesion of lateral popliteal nerve, unspecified lower limb
G57.31	Lesion of lateral popliteal nerve, right lower limb
G57.32	Lesion of lateral popliteal nerve, left lower limb
G57.33	Lesion of lateral popliteal nerve, bilateral lower limbs
G57.4	Lesion of medial popliteal nerve
G57.40	Lesion of medial popliteal nerve, unspecified lower limb
G57.41	Lesion of medial popliteal nerve, right lower limb
G57.42	Lesion of medial popliteal nerve, left lower limb
G57.43	Lesion of medial popliteal nerve, bilateral lower limbs
G57.5	Tarsal tunnel syndrome
G57.50	Tarsal tunnel syndrome, unspecified lower limb
G57.51	Tarsal tunnel syndrome, right lower limb
G57.52	Tarsal tunnel syndrome, left lower limb
G57.53	Tarsal tunnel syndrome, bilateral lower limbs
G57.6	Lesion of plantar nerve
G57.60	Lesion of plantar nerve, unspecified lower limb
G57.61	Lesion of plantar nerve, right lower limb
G57.62	Lesion of plantar nerve, left lower limb
G57.63	Lesion of plantar nerve, bilateral lower limbs
G57.7	Causalgia of lower limb
G57.70	Causalgia of unspecified lower limb
G57.71	Causalgia of right lower limb
G57.72	Causalgia of left lower limb
G57.73	Causalgia of bilateral lower limbs
G57.8	Other specified mononeuropathies of lower limb

© 2022 Hernandez-Romieu AC et al. *JAMA Network Open.*

G57.80	Other specified mononeuropathies of unspecified lower limb
G57.81	Other specified mononeuropathies of right lower limb
G57.82	Other specified mononeuropathies of left lower limb
G57.83	Other specified mononeuropathies of bilateral lower limbs
G57.9	Unspecified mononeuropathy of lower limb
G57.90	Unspecified mononeuropathy of unspecified lower limb
G57.91	Unspecified mononeuropathy of right lower limb
G57.92	Unspecified mononeuropathy of left lower limb
G57.93	Unspecified mononeuropathy of bilateral lower limbs
G58	Other mononeuropathies
G58.0	Intercostal neuropathy
G58.7	Mononeuritis multiplex
G58.8	Other specified mononeuropathies
G58.9	Mononeuropathy, unspecified
G59	Mononeuropathy in diseases classified elsewhere
G61	Inflammatory polyneuropathy
G61.0	Guillain-Barre syndrome
G61.1	Serum neuropathy
G61.8	Other inflammatory polyneuropathies
G61.81	Chronic inflammatory demyelinating polyradiculoneuropathy
G61.82	Multifocal motor neuropathy
G61.89	Other inflammatory polyneuropathies
G61.9	Inflammatory polyneuropathy, unspecified
G62.81	Critial illness polyneuropathy
G62.9	Polyneuropathy, unspecified
G64	Other disorders of the peripheral nervous system
G65	Sequelae of inflammatory and toxic polyneuropathies
G65.0	Sequelae of Guillain-Barre syndrome
G65.1	Sequelae of other inflammatory polyneuropathy
G65.2	Sequelae of toxic polyneuropathy
**Seizures / Status epilepticus / Epilepsy and recurrent seizures**	
G40	Epilepsy and recurrent seizures
G40.0	Localization-related (focal) (partial) idiopathic epilepsy and epileptic syndromes with seizures of localized onset
G40.00	Localization-related (focal) (partial) idiopathic epilepsy and epileptic syndromes with seizures of localized onset, not intractable
G40.001	Localization-related (focal) (partial) idiopathic epilepsy and epileptic syndromes with seizures of localized onset, not intractable, with status epilepticus
G40.009	Localization-related (focal) (partial) idiopathic epilepsy and epileptic syndromes with seizures of localized onset, not intractable, without status epilepticus
G40.01	Localization-related (focal) (partial) idiopathic epilepsy and epileptic syndromes with seizures of localized onset, intractable
G40.011	Localization-related (focal) (partial) idiopathic epilepsy and epileptic syndromes with seizures of localized onset, intractable, with status epilepticus
G40.019	Localization-related (focal) (partial) idiopathic epilepsy and epileptic syndromes with seizures of localized onset, intractable, without status epilepticus

© 2022 Hernandez-Romieu AC et al. *JAMA Network Open.*

G40.1	Localization-related (focal) (partial) symptomatic epilepsy and epileptic syndromes with simple partial seizures
G40.10	Localization-related (focal) (partial) symptomatic epilepsy and epileptic syndromes with simple partial seizures, not intractable
G40.101	Localization-related (focal) (partial) symptomatic epilepsy and epileptic syndromes with simple partial seizures, not intractable, with status epilepticus
G40.109	Localization-related (focal) (partial) symptomatic epilepsy and epileptic syndromes with simple partial seizures, not intractable, without status epilepticus
G40.11	Localization-related (focal) (partial) symptomatic epilepsy and epileptic syndromes with simple partial seizures, intractable
G40.111	Localization-related (focal) (partial) symptomatic epilepsy and epileptic syndromes with simple partial seizures, intractable, with status epilepticus
G40.119	Localization-related (focal) (partial) symptomatic epilepsy and epileptic syndromes with simple partial seizures, intractable, without status epilepticus
G40.2	Localization-related (focal) (partial) symptomatic epilepsy and epileptic syndromes with complex partial seizures
G40.20	Localization-related (focal) (partial) symptomatic epilepsy and epileptic syndromes with complex partial seizures, not intractable
G40.201	Localization-related (focal) (partial) symptomatic epilepsy and epileptic syndromes with complex partial seizures, not intractable, with status epilepticus
G40.209	Localization-related (focal) (partial) symptomatic epilepsy and epileptic syndromes with complex partial seizures, not intractable, without status epilepticus
G40.21	Localization-related (focal) (partial) symptomatic epilepsy and epileptic syndromes with complex partial seizures, intractable
G40.211	Localization-related (focal) (partial) symptomatic epilepsy and epileptic syndromes with complex partial seizures, intractable, with status epilepticus
G40.219	Localization-related (focal) (partial) symptomatic epilepsy and epileptic syndromes with complex partial seizures, intractable, without status epilepticus
G40.3	Generalized idiopathic epilepsy and epileptic syndromes
G40.30	Generalized idiopathic epilepsy and epileptic syndromes, not intractable
G40.301	Generalized idiopathic epilepsy and epileptic syndromes, not intractable, with status epilepticus
G40.309	Generalized idiopathic epilepsy and epileptic syndromes, not intractable, without status epilepticus
G40.31	Generalized idiopathic epilepsy and epileptic syndromes, intractable
G40.311	Generalized idiopathic epilepsy and epileptic syndromes, intractable, with status epilepticus
G40.319	Generalized idiopathic epilepsy and epileptic syndromes, intractable, without status epilepticus
G40.4	Other generalized epilepsy and epileptic syndromes
G40.40	Other generalized epilepsy and epileptic syndromes, not intractable
G40.401	Other generalized epilepsy and epileptic syndromes, not intractable, with status epilepticus
G40.409	Other generalized epilepsy and epileptic syndromes, not intractable, without status epilepticus
G40.41	Other generalized epilepsy and epileptic syndromes, intractable
G40.411	Other generalized epilepsy and epileptic syndromes, intractable, with status epilepticus
G40.419	Other generalized epilepsy and epileptic syndromes, intractable, without status epilepticus
G40.42	Cyclin-Dependent Kinase-Like 5 Deficiency Disorder
G40.5	Epileptic seizures related to external causes
G40.50	Epileptic seizures related to external causes, not intractable
G40.501	Epileptic seizures related to external causes, not intractable, with status epilepticus
G40.509	Epileptic seizures related to external causes, not intractable, without status epilepticus

G40.8	Other epilepsy and recurrent seizures
G40.80	Other epilepsy
G40.801	Other epilepsy, not intractable, with status epilepticus
G40.802	Other epilepsy, not intractable, without status epilepticus
G40.803	Other epilepsy, intractable, with status epilepticus
G40.804	Other epilepsy, intractable, without status epilepticus
G40.81	Lennox-Gastaut syndrome
G40.811	Lennox-Gastaut syndrome, not intractable, with status epilepticus
G40.812	Lennox-Gastaut syndrome, not intractable, without status epilepticus
G40.813	Lennox-Gastaut syndrome, intractable, with status epilepticus
G40.814	Lennox-Gastaut syndrome, intractable, without status epilepticus
G40.82	Epileptic spasms
G40.821	Epileptic spasms, not intractable, with status epilepticus
G40.822	Epileptic spasms, not intractable, without status epilepticus
G40.823	Epileptic spasms, intractable, with status epilepticus
G40.824	Epileptic spasms, intractable, without status epilepticus
G40.83	Dravet syndrome
G40.833	Dravet syndrome, intractable, with status epilepticus
G40.834	Dravet syndrome, intractable, without status epilepticus
G40.89	Other seizures
G40.9	Epilepsy, unspecified
G40.90	Epilepsy, unspecified, not intractable
G40.901	Epilepsy, unspecified, not intractable, with status epilepticus
G40.909	Epilepsy, unspecified, not intractable, without status epilepticus
G40.91	Epilepsy, unspecified, intractable
G40.911	Epilepsy, unspecified, intractable, with status epilepticus
G40.919	Epilepsy, unspecified, intractable, without status epilepticus
G40.A	Absence epileptic syndrome
G40.A0	Absence epileptic syndrome, not intractable
G40.A01	Absence epileptic syndrome, not intractable, with status epilepticus
G40.A09	Absence epileptic syndrome, not intractable, without status epilepticus
G40.A1	Absence epileptic syndrome, intractable
G40.A11	Absence epileptic syndrome, intractable, with status epilepticus
G40.A19	Absence epileptic syndrome, intractable, without status epilepticus
G40.B	Juvenile myoclonic epilepsy [impulsive petit mal]
G40.B0	Juvenile myoclonic epilepsy, not intractable
G40.B01	Juvenile myoclonic epilepsy, not intractable, with status epilepticus
G40.B09	Juvenile myoclonic epilepsy, not intractable, without status epilepticus
G40.B1	Juvenile myoclonic epilepsy, intractable
G40.B11	Juvenile myoclonic epilepsy, intractable, with status epilepticus
G40.B19	Juvenile myoclonic epilepsy, intractable, without status epilepticus
G41	Status epilepticus
G41.0	Grand mal status epilepticus
G41.1	Petit mal status epilepticus
G41.2	Complex partial status epilepticus

G41.8	Other status epilepticus
G41.9	Status epilepticus, unspecified
**Ataxia / Trouble Walking**	
G26	Extrapyramidal and movement disorders in diseases classified elsewhere
R26	Abnormalities of gait and mobility
R26.0	Ataxic gaint
R26.1	Paralytic gait
R26.2	Difficulty in walking, not elsewhere classified
R26.3	Immobility (bedfast, chairfast)
R26.8	Other and unspecified abnormatlities of gait and mobility (unsteadiness on feet NOS)
R26.81	Unsteadiness on feet
R26.89	Other abnormalities of gait and mobility
R26.9	Unspecified abnormalities of gait and mobility
R27.0	Ataxia, unspecified
R27.8	Other and unspecified lack of coordination
R27.9	Repeated falls
R28.2	Difficulty in walking, not elsewhere classified
**Type 2 Diabetes**	
E11	TYPE 2 DIABETES MELLITUS
E11.0	TYPE 2 DIABETES MELLITUS WITH HYPEROSMOLARITY
E11.00	TYPE 2 DM W/HYPEROSMOLARITY W/O NKHHC
E11.01	TYPE 2 DIAB MELLITUS W/HYPEROSMOLARITY W/COMA
E11.1	TYPE 2 DIABETES MELLITUS WITH KETOACIDOSIS
E11.10	TYPE 2 DIABETES MELLITUS W/KETOACIDOSIS W/O COMA
E11.11	TYPE 2 DIABETES MELLITUS WITH KETOACIDOSIS COMA
E11.2	TYPE 2 DIABETES MELLITUS W/KIDNEY COMPLICATIONS
E11.21	TYPE 2 DIABETES MELLITUS W/DIABETIC NEPHROPATHY
E11.22	TYPE 2 DIABETES MELLITUS W/DIAB CHRON KIDNEY DZ
E11.29	TYPE 2 DIABETES MELLITUS W/OTH DIAB KIDNEY COMP
E11.3	TYPE 2 DIABETES MELLITUS W/OPHTHALMIC COMP
E11.31	TYPE 2 DIABETES MELLITUS W/UNS DIAB RETINOPATHY
E11.311	TYPE 2 DM W/UNS DIAB RETINPATHY W/MACULAR EDEMA
E11.319	TYPE 2 DM W/UNS DIAB RETINPATH W/O MACULAR EDEMA
E11.32	TYPE 2 DM W/MILD NONPROLIF DIABETIC RETINPATHY
E11.321	TYPE 2 DM W/MILD NONPROLIF DIAB RETINOPATHY W/ME
E11.3211	TYPE 2 DIABETES MELLITUS MILD NPD MACULAR ED OD
E11.3212	TYPE 2 DIABETES MELLITUS MILD NPD MACULAR ED OS
E11.3213	TYPE 2 DIABETES MELLITUS MILD NPD MACULAR ED BIL
E11.3219	TYPE 2 DIAB MELLITUS MILD NPD MACULAR ED UNS EYE
E11.329	TYPE 2 DM W/MILD NONPROLIF DIAB RETINPATH W/O ME
E11.3291	TYPE 2 DIABETES MELLITUS MILD NPDR W/O MAC ED OD
E11.3292	TYPE 2 DIABETES MELLITUS MILD NPDR W/O MAC ED OS
E11.3293	TYPE 2 DIABETES MELLITUS MILD NPDR W/O MAC ED BL
E11.3299	TYPE 2 DIABETES MELLITUS MLD NPDR W/O ME UNS EYE
E11.33	TYPE 2 DM W/MOD NONPROLIF DIABETIC RETINOPATHY

© 2022 Hernandez-Romieu AC et al. *JAMA Network Open.*

E11.331	TYPE 2 DM W/MOD NONPROLIF DIAB RETINOPATHY W/ME
E11.3311	TYPE 2 DIABETES MELLITUS MOD NPDR MACULAR ED OD
E11.3312	TYPE 2 DIABETES MELLITUS MOD NPDR MACULAR ED OS
E11.3313	TYPE 2 DIABETES MELLITUS MOD NPDR MACULAR ED BIL
E11.3319	TYPE 2 DIABETES MELLITUS MOD NPDR MAC ED UNS EYE
E11.339	TYPE 2 DM W/MOD NONPROLIF DM RETINOPATHY W/O ME
E11.3391	TYPE 2 DIABETES MELLITUS MOD NPDR W/O MAC ED OD
E11.3392	TYPE 2 DIABETES MELLITUS MOD NPDR W/O MAC ED OS
E11.3393	TYPE 2 DIABETES MELLITUS MOD NPDR W/O MAC ED BIL
E11.3399	TYPE 2 DIABETES MELLITUS MOD NPDR W/O ME UNS EYE
E11.34	TYPE 2 DM W/SEVERE NONPROLIF DIAB RETINOPATHY
E11.341	TYPE 2 DM W/SEV NONPROLIF DIAB RETINOPATHY W/ME
E11.3411	TYPE 2 DIABETES MELLITUS SVR NPDR MACULAR ED OD
E11.3412	TYPE 2 DIABETES MELLITUS SVR NPDR MACULAR ED OS
E11.3413	TYPE 2 DIABETES MELLITUS SVR NPDR MACULAR ED BIL
E11.3419	TYPE 2 DIABETES MELLITUS SVR NPDR MAC ED UNS EYE
E11.349	TYPE 2 DM W/SEV NONPROLIF DIAB RETINOPATH W/O ME
E11.3491	TYPE 2 DIABETES MELLITUS SVR NPDR W/O MAC ED OD
E11.3492	TYPE 2 DIABETES MELLITUS SVR NPDR W/O MAC ED OS
E11.3493	TYPE 2 DIABETES MELLITUS SVR NPDR W/O MAC ED BIL
E11.3499	TYPE 2 DIABETES MELLITUS SVR NPDR W/O ME UNS EYE
E11.35	TYPE 2 DM W/PROLIFERATIVE DIABETIC RETINOPATHY
E11.351	TYPE 2 DM W/PROLIFERATIVE DIAB RETINOPATHY W/ME
E11.3511	TYPE 2 DIABETES MELLITUS PDR MACULAR EDEMA OD
E11.3512	TYPE 2 DIABETES MELLITUS PDR MACULAR EDEMA OS
E11.3513	TYPE 2 DIABETES MELLITUS PDR MACULAR EDEMA BILAT
E11.3519	TYPE 2 DIABETES MELLITUS PDR MACULAR ED UNS EYE
E11.359	TYPE 2 DM PROLIF DM RETINOPATHY NO MACULAR EDEMA
E11.3591	TYPE 2 DIABETES MELLITUS PDR W/O MACULAR ED OD
E11.3592	TYPE 2 DIABETES MELLITUS PDR W/O MACULAR ED OS
E11.3593	TYPE 2 DIABETES MELLITUS PDR W/O MACULAR ED BIL
E11.3599	TYPE 2 DIABETES MELLITUS PDR W/O MAC ED UNS EYE
E11.36	TYPE 2 DIABETES MELLITUS WITH DIABETIC CATARACT
E11.37	TYPE 2 DIAB MELLITUS DIAB MAC EDEMA RSLVD FLW TX
E11.37X1	TYPE 2 DIABETES MELLITUS DIAB ME RSLVD FLW TX OD
E11.37X2	TYPE 2 DIABETES MELLITUS DIAB ME RSLVD FLW TX OS
E11.37X3	TYPE 2 DIABETES MELLITUS DIAB ME RSLVD FLW TX BL
E11.37X9	TYPE 2 DIAB MELLITUS DB ME RSLVD FLW TX UNS EYE
E11.39	TYPE 2 DIABETES MELLITUS OTH DIAB OPHTHALM COMP
E11.4	TYPE 2 DIABETES MELLITUS W/NEUROLOGICAL COMP
E11.40	TYPE 2 DM WITH DIABETIC NEUROPATHY UNSPECIFIED
E11.41	TYPE 2 DIABETES MELLITUS W/DIAB MONONEUROPATHY
E11.42	TYPE 2 DIABETES MELLITUS W/DIAB POLYNEUROPATHY
E11.43	TYPE 2 DM W/DIABETIC AUTONOMIC POLYNEUROPATHY
E11.44	TYPE 2 DIABETES MELLITUS W/DIABETIC AMYOTROPHY

© 2022 Hernandez-Romieu AC et al. *JAMA Network Open.*

E11.49	TYPE 2 DIABETES MELLITUS W/OTH DIAB NEURO COMP
E11.5	TYPE 2 DIABETES MELLITUS W/CIRCULATORY COMP
E11.51	TYPE 2 DM W/DIAB PERIPH ANGIOPATHY W/O GANGRENE
E11.52	TYPE 2 DM W/DIAB PERIPH ANGIOPATHY W/GANGRENE
E11.59	TYPE 2 DIABETES MELLITUS W/OTH CIRCULATORY COMP
E11.6	TYPE 2 DIABETES MELLITUS W/OTH SPEC COMPLICATION
E11.61	TYPE 2 DIABETES MELLITUS W/DIABETIC ARTHROPATHY
E11.610	TYPE 2 DM W/DIABETIC NEUROPATHIC ARTHROPATHY
E11.618	TYPE 2 DIABETES MELLITUS W/OTH DIAB ARTHROPATHY
E11.62	TYPE 2 DIABETES MELLITUS WITH SKIN COMPLICATIONS
E11.620	TYPE 2 DIABETES MELLITUS W/DIABETIC DERMATITIS
E11.621	TYPE 2 DIABETES MELLITUS WITH FOOT ULCER
E11.622	TYPE 2 DIABETES MELLITUS WITH OTHER SKIN ULCER
E11.628	TYPE 2 DIABETES MELLITUS W/OTH SKIN COMP
E11.63	TYPE 2 DIABETES MELLITUS WITH ORAL COMPLICATIONS
E11.630	TYPE 2 DIABETES MELLITUS W/PERIODONTAL DISEASE
E11.638	TYPE 2 DIABETES MELLITUS W/OTH ORAL COMP
E11.64	TYPE 2 DIABETES MELLITUS WITH HYPOGLYCEMIA
E11.641	TYPE 2 DIABETES MELLITUS W/HYPOGLYCEMIA W/COMA
E11.649	TYPE 2 DIABETES MELLITUS W/HYPOGLYCEMIA W/O COMA
E11.65	TYPE 2 DIABETES MELLITUS WITH HYPERGLYCEMIA
E11.69	TYPE 2 DIABETES MELLITUS W/OTH SPEC COMPLICATION
E11.8	TYPE 2 DIABETES MELLITUS W/UNS COMPLICATIONS
E11.9	TYPE 2 DIABETES MELLITUS WITHOUT COMPLICATIONS

eTable 2: Institutions contributing data

PCORnet Clinical Research Network	Healthcare system
STAR	Duke University
STAR	Medical University of SC
STAR	University of North Carolina
STAR	Vanderbilt University Medical Center
STAR	Wake Forest Baptist Health
GPC	Allina Health
GPC	Intermountain HC
GPC	Medical College of Wisconsin
GPC	University of Iowa Healthcare
GPC	University of Kansas
GPC	University of Nebraska
GPC	University of Texas SW Medical Center
GPC	University of Utah
REACHnet	UMC New Orleans
PEDsNet	Children's Hospital Colorado
PEDsNet	Children's Hospital of Philadelphia
PEDsNet	Cincinnati Children's Hospital
PEDsNet	Nationwide Children's Hospital
PEDsNet	Nemours Children's Hospital
PEDsNet	St. Louis Children's Hospital
INSIGHT	Montefiore
INSIGHT	Mount Sinai Health System
INSIGHT	NYU Langone Medical Center
INSIGHT	Weill Cornell Medicine
CAPriCORN	Cook County
CAPriCORN	Lurie Children's Hospital
CAPriCORN	Northwestern University
ADVANCE	Fenway Health
ADVANCE	Health Choice Network
ADVANCE	OCHIN
PaTH	Johns Hopkins University
PaTH	Ohio State University
PaTH	Penn State College of Medicine and Penn State Health Milton S. Hershey Medical Center
PaTH	Temple University
PaTH	University of Michigan
PaTH	UPMC
OneFlorida	AdventHealth
OneFlorida	Orlando Health System
OneFlorida	UF Health
OneFlorida	University of Miami

© 2022 Hernandez-Romieu AC et al. *JAMA Network Open.*

eTable 3. Prevalence of new diagnoses of symptoms and conditions among adults and children and young adults with positive or negative SARS-CoV-2 test results with medical encounters 31 to 150 days after first test, PCORnet, March to December 2020.

	Non-hospitalized			Hospitalized			Mechanically Ventilated		
	SARS-CoV-2 positive n/total† (%)	SARS-CoV-2 negative n/total† (%)	Unadjusted Prevalence Ratio* Est. (99%CI)	SARS-CoV-2 positive n/total† (%)	SARS-CoV-2 negative n/total† (%)	Unadjusted Prevalence Ratio* Est. (99%CI)	SARS-CoV-2 positive n/total† (%)	SARS-CoV-2 negative n/total† (%)	Unadjusted Prevalence Ratio* Est. (99%CI)
Adults: Incident symptoms and conditions in the 31–150 days following SARS-CoV-2 test									
Symptoms									
Shortness of Breath	5703/128251 (4.45)	43208/1061692 (4.07)	1.09 (1.05, 1.13)	1932/18419 (10.49)	17782/320902 (5.54)	1.89 (1.79, 2.01)	186/1123 (16.56)	896/9166 (9.78)	1.69 (1.40, 2.05)
Fatigue	5300/125749 (4.21)	47017/1061342 (4.43)	0.95 (0.92, 0.99)	1570/19677 (7.98)	19790/334893 (5.91)	1.35 (1.27, 1.44)	245/1236 (19.82)	1083/10825 (10.00)	1.98 (1.68, 2.34)
Sleep Disorders	3857/122613 (3.15)	38835/1013807 (3.83)	0.82 (0.79, 0.86)	1058/18821 (5.62)	16124/322673 (5.00)	1.12 (1.04, 1.22)	118/1136 (10.39)	937/10072 (9.30)	1.12 (0.88, 1.42)
Headache	3682/131406 (2.80)	33758/1125058 (3.00)	0.93 (0.89, 0.98)	517/22162 (2.33)	8841/366957 (2.41)	0.97 (0.86, 1.09)	28/1404 (1.99)	323/12716 (2.54)	0.79 (0.48, 1.30)
Heart Rate Abnormality§	3509/133276 (2.63)	29543/1114330 (2.65)	0.99 (0.95, 1.04)	1102/20594 (5.35)	14815/347379 (4.26)	1.25 (1.16, 1.36)	127/1303 (9.75)	865/10964 (7.89)	1.24 (0.98, 1.56)
Cognitive dysfunction	3267/131245 (2.49)	31989/1099309 (2.91)	0.86 (0.82, 0.90)	1102/19930 (5.53)	16247/343265 (4.73)	1.17 (1.08, 1.26)	124/1251 (9.91)	949/11129 (8.53)	1.16 (0.92, 1.47)
Change in Bowel Habits	2733/132350 (2.06)	29966/1103068 (2.72)	0.76 (0.72, 0.80)	879/20657 (4.26)	15365/346939 (4.43)	0.96 (0.88, 1.05)	96/1297 (7.40)	741/11453 (6.47)	1.14 (0.87, 1.50)
Weight Loss	1132/140719 (0.80)	16511/1177543 (1.40)	0.57 (0.53, 0.62)	848/22331 (3.80)	13605/367617 (3.70)	1.03 (0.94, 1.12)	167/1371 (12.18)	1034/11757 (8.79)	1.39 (1.13, 1.69)
Sensation and Perception	1191/139605 (0.85)	13089/1175316 (1.11)	0.77 (0.71, 0.83)	329/22566 (1.46)	5806/375507 (1.55)	0.94 (0.82, 1.09)	25/1420 (1.76)	253/12662 (2.00)	0.88 (0.52, 1.50)
Conditions									
Anxiety/Depression	3934/119625 (3.29)	40397/982068 (4.11)	0.8 (0.77, 0.83)	930/19218 (4.84)	17428/318711 (5.47)	0.88 (0.81, 0.96)	114/1197 (9.52)	885/10477 (8.45)	1.13 (0.88, 1.44)
Type 2 Diabetes	1999/120706 (1.66)	19012/1038132 (1.83)	0.9 (0.85, 0.96)	1140/15740 (7.24)	11179/312623 (3.58)	2.03 (1.87, 2.19)	154/921 (16.72)	708/9509 (7.45)	2.25 (1.82, 2.77)
Peripheral Nerve Disorders	1645/136554 (1.20)	18258/1143447 (1.60)	0.75 (0.71, 0.81)	461/21948 (2.10)	8049/364039 (2.21)	0.95 (0.84, 1.07)	99/1381 (7.17)	432/12345 (3.50)	2.05 (1.55, 2.70)
Ataxia/Trouble Walking	1004/140466 (0.71)	12979/1177461 (1.10)	0.65 (0.60, 0.71)	496/22218 (2.23)	8131/370308 (2.20)	1.02 (0.90, 1.14)	102/1398 (7.30)	443/12493 (3.55)	2.06 (1.57, 2.70)
Autonomic Dysfunction	491/142854 (0.34)	5928/1205120 (0.49)	0.7 (0.62, 0.79)	231/23265 (0.99)	4133/383802 (1.08)	0.92 (0.78, 1.10)	24/1459 (1.64)	230/12897 (1.78)	0.92 (0.53, 1.60)
Seizures	251/142809 (0.18)	3498/1206410 (0.29)	0.61 (0.51, 0.72)	208/23143 (0.90)	3091/383098 (0.81)	1.11 (0.93, 1.34)	21/1418 (1.48)	378/12656 (2.99)	0.50 (0.28, 0.88)
Myoneural Disorders	151/144129 (0.10)	1556/1220689 (0.13)	0.82 (0.66, 1.02)	148/23667 (0.63)	1157/391284 (0.30)	2.11 (1.69, 2.65)	83/1455 (5.70)	130/13184 (0.99)	5.79 (4.06, 8.25)
Children/young adults: Incident symptoms and conditions in the 31–150 days following SARS-CoV-2 test									
Symptoms									
Change in Bowel Habits	523/22999 (2.27)	6010/231668 (2.59)	0.88 (0.78, 0.98)	62/1040 (5.96)	2253/41498 (5.43)	1.10 (0.80, 1.51)	---	---	---
Fatigue	405/24238 (1.67)	4033/249755 (1.61)	1.03 (0.91, 1.18)	42/1217 (3.45)	1191/47439 (2.51)	1.37 (0.92, 2.05)	---	---	---
Shortness of Breath	426/24049 (1.77)	3433/244651 (1.40)	1.26 (1.11, 1.44)	46/1179 (3.90)	1048/46092 (2.27)	1.72 (1.17, 2.51)	---	---	---
Headache	436/24146 (1.81)	4303/251359 (1.71)	1.05 (0.93, 1.20)	19/1267 (1.50)	801/48392 (1.66)	0.91 (0.50, 1.64)	---	---	---
Heart Rate Abnormality§	251/24545 (1.02)	2248/250999 (0.90)	1.14 (0.96, 1.35)	49/1139 (4.30)	1353/46288 (2.92)	1.47 (1.02, 2.12)	---	---	---
Cognitive dysfunction	283/24388 (1.16)	2638/251165 (1.05)	1.1 (0.94, 1.30)	27/1243 (2.17)	880/47769 (1.84)	1.18 (0.72, 1.94)	---	---	---
Weight Loss	169/24586 (0.69)	1959/250073 (0.78)	0.88 (0.71, 1.08)	34/1191 (2.85)	1330/46225 (2.88)	0.99 (0.64, 1.54)	---	---	---
Sensation and Perception	194/24603 (0.79)	2969/248714 (1.19)	0.66 (0.55, 0.80)	17/1230 (1.38)	1143/46891 (2.44)	0.57 (0.30, 1.06)	---	---	---
Conditions									
Anxiety/Depression	584/23016 (2.54)	5196/240107 (2.16)	1.17 (1.05, 1.31)	53/1152 (4.60)	1772/45258 (3.92)	1.18 (0.83, 1.67)	---	---	---
Seizures	36/24874 (0.14)	944/252135 (0.37)	0.39 (0.25, 0.60)	17/1203 (1.41)	899/45667 (1.97)	0.72 (0.38, 1.34)	---	---	---
Autonomic Dysfunction	46/25195 (0.18)	574/258318 (0.22)	0.82 (0.55, 1.22)	10/1309 (0.76)	364/49574 (0.73)	1.04 (0.46, 2.37)	---	---	---
Diabetes Type 2	27/25187 (0.11)	220/259712 (0.08)	1.27 (0.75, 2.14)	17/1293 (1.31)	308/50207 (0.61)	2.14 (1.13, 4.06)	---	---	---

CI = confidence interval

* Unadjusted prevalence ratio was calculated as follows: %PCC among SARS-CoV-2 positive/%PCC among SARS-CoV-2 negative with 99% confidence intervals = ; 99% confidence intervals = e^($\{\ln(PR) \pm [2.576 * SE(\ln(PR))]\}$)

†n = persons with symptom or conditions diagnosed in 31–150 days after SARS-CoV-2 testing who did not have the condition recorded in the 18 months to 7 days prior to SARS-CoV-2 testing; total = number of persons with medical encounters 31–150 days after SARS-CoV-2 testing minus the number of persons with medical encounters 31–150 days who had the symptom or condition recorded in the 18 months to 7 days prior to SARS-CoV-2 testing. Total denominators are different for each row due to removal of persons with symptoms and conditions in the baseline period.

§Nonspecific heart rate abnormalities include tachycardia, bradycardia, and palpitations.

© 2022 Hernandez-Romieu AC et al. *JAMA Network Open.*

© 2022 Hernandez-Romieu AC et al. *JAMA Network Open.*
